# Metabolic programming defines oxygen-sensitive subpopulation hierarchies and patterning in collective invasion

**DOI:** 10.1091/mbc.E25-07-0314

**Published:** 2025-10-16

**Authors:** Veronika Y. Matsuk, Tala O. Khatib, Landon J. Marcus, Isaac E. Robinson, Yuan Liu, Janani K. Pasupathy, Mala Shanmugam, Janna K. Mouw, Adam I. Marcus

**Affiliations:** a Department of Hematology and Medical Oncology, Emory University School of Medicine, Atlanta, GA 30322; b Winship Cancer Institute of Emory University, Atlanta, GA 30322; c Graduate Program in Cancer Biology, Emory University, Atlanta, GA 30322; d Graduate Program in Biochemistry, Cell, and Developmental Biology, Emory University, Atlanta, GA 30322; e Fred Hutchinson Cancer Center, Seattle, WA 98109; f Community Scientist, Atlanta, GA 30033; g George W. Woodruff School of Mechanical Engineering, Georgia Institute of Technology, Atlanta, GA 30332; h Department of Biostatistics and Bioinformatics, Rollins School of Public Health, Emory University, Atlanta, GA 30322; i Emory Integrated Computational Core, Emory University, Atlanta, GA 30322; Reed College

## Abstract

Phenotypic heterogeneity—distinct molecular and behavioral variations within a population—significantly influences collective invasion and tumor progression. Here, we use a molecular approach to explore how the underlying metabolic heterogeneity in non-small cell lung cancer (NSCLC) influences invasion and pack patterning. Assessment of  three-dimensional (3D) pack patterning revealed invasive heterogeneity across NSCLC cell lines and patient-derived samples. Flow cytometry identified IL13RA2 as a biomarker for invasive potential, enabling isolation of subpopulations with distinct invasive characteristics. By integrating a cell surface biomarker (IL13RA2±) with mitochondrial membrane potential (TMRM), we identified and isolated three distinct subpopulations. Two-dimensional (2D) analyses revealed differences in mitochondrial polarity and transcriptional programs associated with migration and oxygensensitivity. In 3D, these subpopulations invaded with distinct patterns, from contiguous circular packs to structured chains. Assessments under varied oxygen tension demonstrated that oxygen availability and subpopulation metabolism together influence collective invasion patterning. When recombined at ratios recapitulating the original population, both stochastic and opportunistic cooperative dynamics emerged, dependent on subpopulation composition and oxygen levels. Our molecular approach, integrating cell surface and metabolic characteristics, enables the isolation of unique subpopulations and demonstrates that phenotypic and metabolic heterogeneity, population composition, and oxygen availability collectively pattern invasion packs and drive collective invasion.

## Abstract (executive summary)

- Tumor cell populations exhibit phenotypic and metabolic heterogeneity, which is thought to influence collective invasion and cancer progression, but the underlying mechanisms remain unclear.
- Using molecular and metabolic markers, this study isolated distinct subpopulations of NSCLC cells, revealing that differences in metabolism and oxygen sensitivity drive unique invasion patterns and cooperative behaviors.
- These findings reveal that both intrinsic cellular heterogeneity and extrinsic microenvironmental factors integrate to drive collective invasion, advancing our understanding of the cooperative cellular behaviors underlying this process.

## INTRODUCTION

Recent studies suggest that non–small cell lung cancer (NSCLC) can spread into the surrounding lung stroma through a process known as collective invasion. This is a collaborative mechanism where heterogeneous subpopulations move together as a cohesive unit, maintaining cell–cell junctions (Van Zijl *et al.*, 2011; Friedl *et al.*, 2012; Chaffer *et al.*, 2016; Keller and Pantel, 2019; Yang *et al.*, 2019; Vilchez Mercedes *et al.*, 2021; Hapach *et al.*, 2023; Zhang and Reinhart-King, 2023). These collectively invading cells have been observed in patient samples (Gilbert-Ross *et al.*, 2017), mouse models (Matise *et al.*, 2012; Ilina *et al.*, 2018), and within three-dimensional (3D) in vitro environments (Carey *et al.*, 2013; Konen *et al.*, 2017; Zhang *et al.*, 2019; Commander *et al.,* 2020; Summerbell *et al.*, 2020; Khalil *et al.*, 2024; Khatib *et al.*, 2025). In 3D lung cancer models, collectively invading cells can maintain a chain-like invasive pattern characterized by distinct localization and cellular roles within a pack. Using a live cell isolation technique that our lab developed to investigate 3D collective invasion, we isolated stable subpopulations based on their location within actively invading packs (Konen *et al.*, 2017; Khatib *et al.*, 2023). These subpopulations—leaders that drive pack invasion and followers that trail behind and comprise the majority—maintain their respective invasive properties and have multiple levels of heterogeneity with distinct genetic (Zoeller *et al.*, 2019; Pedro *et al.*, 2020), transcriptional (Sharma *et al.*, 2024), epigenetic (Summerbell *et al.*, 2020), and metabolic (Commander *et al.*, 2020) profiles.

Cooperative collective invasion drives tumor progression more efficiently when compared with single-cell invasion (Shin *et al.*, 2014; Nagai *et al.*, 2020) and, therefore, studies investigating the underlying phenotypic heterogeneity have garnered interest. Our laboratory has demonstrated that, within the NSCLC collectively invading pack, leaders are heavily dependent on oxidative phosphorylation, and followers preferentially utilize glycolysis (Commander *et al.,* 2020). When we pharmacologically target these different metabolic phenotypes, either alone or in combination (Commander *et al.,* 2020), collective invasion is inhibited (Knippler *et al.*, 2024)—illustrating the metabolic sensitivities underlying collective invasion. Ultimately, the nutrient microenvironment determines the impact of this metabolic heterogeneity and, thus, the metabolic vulnerability. NSCLC originates within the oxygen-rich lung environment (Warburg *et al.*, 1927; Helmlinger *et al.*, 1997; Dang and Semenza, 1999; Gatenby and Gillies, 2004; Heiden *et al.*, 2009). However, due to its poor diffusion, oxygen levels are dramatically reduced in NSCLC primary tumors (Wild *et al.*, 2005; Le *et al.*, 2006; Miller *et al.*, 2010).

To further deconstruct how underlying metabolic heterogeneity drives collective invasion in a nutrient- and oxygen-dependent manner, we designed an approach, incorporating metabolic profiling to detect subpopulations. This was done by integrating cell surface biomarkers, identified from previously isolated subpopulations, with metabolic profiling of mitochondrial polarity, which corresponds with sensitivity to microenvironmental oxygen levels. Our methodology identified three distinct subpopulations of collectively invading cells, each with unique metabolic, transcriptional, and invasive characteristics in isolation. We found that these subpopulations demonstrated unique cooperative dynamics during invasion dictated by both oxygen availability and composition, with these behaviors driven by distinct underlying mechanisms. Our findings underscore that the mechanisms driving collective invasion are both cell intrinsic (metabolic, invasive, and transcriptional heterogeneity) and extrinsic (oxygen availability within the microenvironment).

## RESULTS

### NSCLC cell lines and patient samples exhibit molecular and phenotypic heterogeneity

#### NSCLC cell lines and patient samples exhibit distinct invasive phenotypes

**FIGURE 1:**
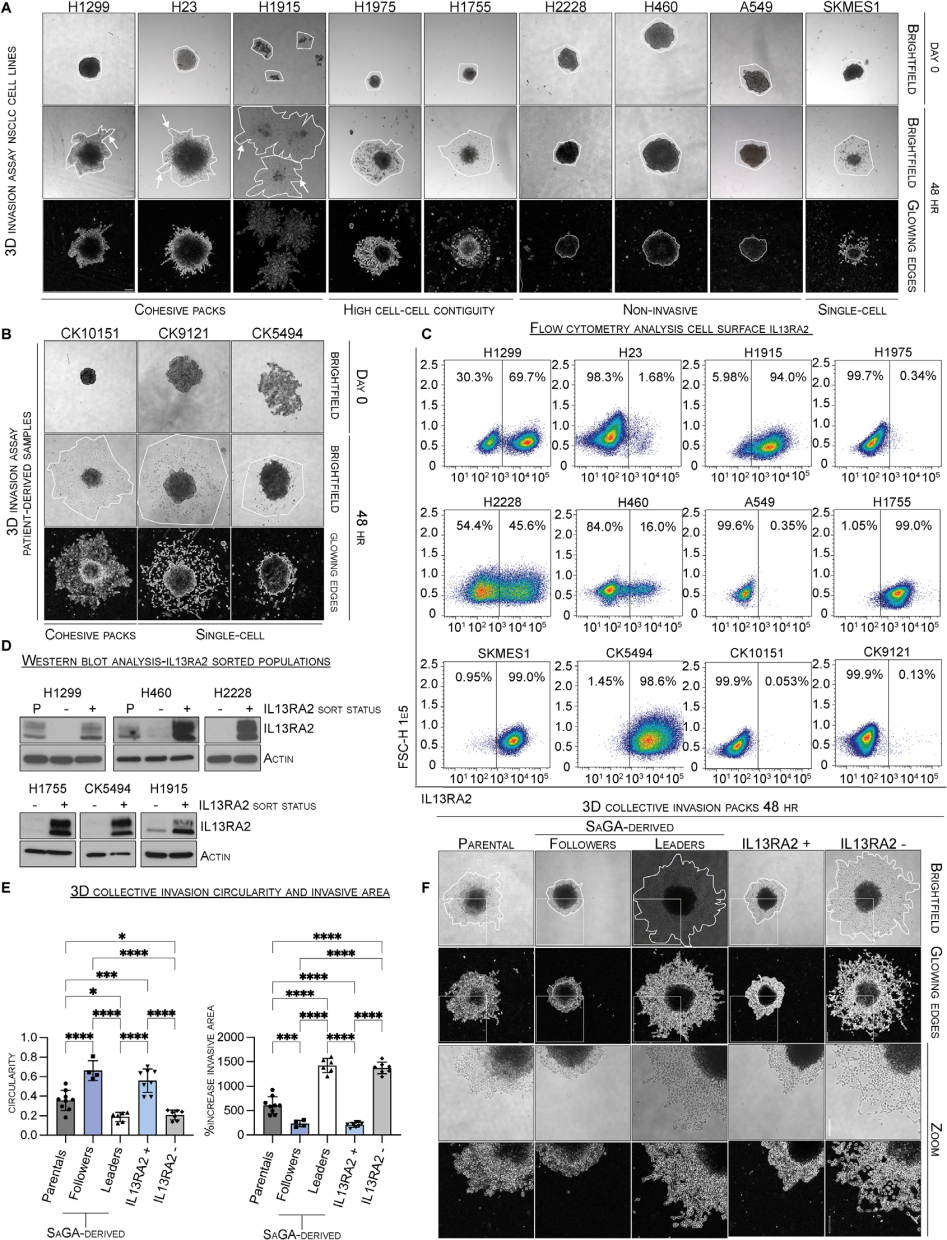
NSCLC cell lines and patient samples exhibit molecular and phenotypic heterogeneity. (A and B) Spheroids composed of NSCLC cell lines or patient-derived samples were embedded in Matrigel and allowed to invade for 48 h. Brightfield representative images are shown on day 0 and 48 h. Solid (white) lines outline the outer perimeter of the invasive area after 48 h; scale bar, 200 µm. (*n* = 5 H1299) (*n* = 5 H23) (*n* = 3 H1915) (*n* = 3 H1975) (*n* = 2 H1755) (*n* = 1 H2228) (*n* = 3 A549) (*n* = 3 H460) (*n* = 2 SKMES1) (*n* = 1 CK10151,CK9121,CK5494). (C) Flow cytometry plots illustrating IL13RA2 expression in NSCLC cell lines and patient-derived samples. (*n* = 4 H1299) (*n* = 2 H23) (*n* = 2 H1915) (*n* = 2 H1975) (*n* = 1 H1755, H2228, A549,H460, CK10151,CK9121,CK5494) (*n* = 2 SKMES1) (D) Immunoblotting of H1299, H460, H2228, H1755, CK5494, and H1915 cell lines following FACS sorting on IL13RA2 expression. Actin was used as a loading control (*n* = 1). (E) Quantification of overall circularity and percent increase in invasive area after 48 h of invasion in Matrigel. To assess significance, an ordinary one-way ANOVA with a Tukey's multiple comparisons test was used, ∗*p* ≤ 0.05, ∗∗*p* ≤ 0.01, ∗∗∗*p* ≤ 0.001, ∗∗∗∗*p* ≤ 0.0001.) (F) Brightfield representative images are shown of Parental, SaGA-derived followers and leaders, IL13RA2^+^-sorted population, and IL13RA2^−^-sorted population at 48 h; scale bar, 200 µm. Mean ± SEM is shown (*n* = 1) and (*N* = 9 Parentals) (*N* = 4 SaGA-derived Followers) (*N* = 6 SaGA-derived Leaders) (*N* = 8 IL13RA2 positive) (*N* = 7 IL13RA2 negative). See also Supplemental Figure S1.

We hypothesized that NSCLC cell lines and patient-derived samples would exhibit distinct invasive phenotypes reflective of  underlying molecular heterogeneity. To investigate invasive heterogeneity in NSCLC, we used a panel of NSCLC cell lines and patient-derived samples. During our investigation of 3D spheroid collective invasion, we found that the H2228, H460, and A549 cell lines did not invade within the recombinant basement membrane (rBM, Matrigel) matrix (Figure 1A). In contrast, the H1299, H23, H1915, H1975, H1755, and SKMES1 cell lines, and the patient-derived CK10151, CK9121, and CK5494 lines, displayed heterogeneous invasive phenotypes.

Specifically, the H23, H1915, and CK10151 lines exhibited collective invasion patterning similar to our H1299 model cell line (Konen *et al.*, 2017; Khatib *et al.*, 2023; 2025) (Figure 1, A and B). These cells formed clear, cohesive packs of invading cells with a series of follower cells trailing behind the leader cells. In contrast, the H1975, H1755, and H2228 lines lacked distinct packs and instead exhibited high cell–cell contiguity invasion patterns with high circularity at the invasive edges (Figure 1A). The SKMES1, CK9121, and CK5494 lines lacked distinct collective invasion packs, instead invading as single cells into the surrounding matrix, suggesting an absence of cell–cell junctions (Figure 1, A and B).

Within this analysis, cohesive packs were defined as discrete multicellular invasive projections with leader and follower organization. Invasion lacking distinct projections was characterized by continuous cell–cell contacts without a pack structure, instead forming sheet-like fronts. These findings highlight  invasive heterogeneity within and across NSCLC cell lines and patient-derived samples, suggesting diverse underlying molecular mechanisms that define these invasive patterns. These observations revealed whether NSCLC cell lines and patient samples rely on cohesive collective invasion packs or single-cell dispersal, informing us on the likely molecular programs driving each phenotype.

#### IL13RA2 identifies molecularly and functionally distinct invasive subpopulations in NSCLC

Previously, we identified IL13RA2 as a cell surface biomarker for follower cells in our H1299 model cell line (Summerbell *et al.*, 2020). As such, we conducted a pairwise comparison of IL13RA2 mRNA expression using our SaGA-derived populations. SaGA (Spatiotemporal Genomic and Cellular Analysis) enables the isolation of spatially distinct subpopulations, such as SaGA-derived leaders and SaGA-derived followers, within collectively invading packs. Our results consistent with our previous findings, demonstrating that IL13RA2 was significantly upregulated in follower cells when compared with leader cells, exhibiting a fold-change of 13 and a *p*-value less than 0.0005 (Supplemental Figure S1, A and B). We validated these transcriptional data using two-dimensional (2D) whole-cell lysates (Supplemental Figure S1C), which revealed that follower cells exhibited the highest levels of IL13RA2 expression, intermediate levels were found in the parental cells, while leader cells showed no detectable expression.

To investigate the broader relevance of IL13RA2 in NSCLC, we used flow cytometry to evaluate IL13RA2 cell surface expression across multiple NSCLC cell lines and patient-derived samples. Flow cytometry analysis of cell surface IL13RA2 biomarker expression in the parental H1299 cell line revealed that ∼30% of the cells were IL13RA2-negative (IL13RA2^−^), while ∼70% were IL13RA2-positive (IL13RA2^+^) (Figure 1C). This biomarker heterogeneity extended beyond the H1299 parental cell line (Supplemental Figure S1D). The H1915, H2228, and H460 cell lines exhibited a biphasic distribution of IL13RA2, while the H1755, SKMES1, and CK5494 lines were predominantly composed of IL13RA2^+^ cells (Figure 1C; Supplemental Figure S1D). In contrast, the A549, H23, H1975, CK10151, and CK9121 lines were primarily IL13RA2^−^ cells (Figure 1C; Supplemental Figure S1D). These results supported IL13RA2 cell surface heterogeneity across NSCLC cell lines and are consistent with observed invasive heterogeneity.

Because we previously identified IL13RA2 as a follower biomarker in our H1299 model cell line (Summerbell *et al.*, 2020), we hypothesized that subpopulations isolated based on cell-surface IL13RA2 expression would phenocopy the invasive signatures of SaGA-derived leaders and followers. By utilizing fluorescence-activated cell sorting (FACS), we isolated live IL13RA2^−^ and IL13RA2^+^ populations from the H1299, H460, H2228, H1755, CK5494, and H1915 cell lines (Figure 1D). 2D whole-cell lysates from isolated IL13RA2^−^ and IL13RA2^+^ subpopulations confirmed the IL13RA2 protein heterogeneity observed via flow cytometry (Figure 1D).

Functionally, the subpopulations (IL13RA2^+^ and IL13RA2^−^) sorted from the H1299 model cell line exhibited distinct invasive capabilities in isolation (Figure 1, E and F). IL13RA2^+^ cells had a high contiguity/high circularity invasion pattern that lacked distinct and individual collective invasion packs, phenocopying the SaGA-derived followers (Figure 1F). Although IL13RA2^−^ spheroids were significantly more invasive than their IL13RA2^+^ counterparts, their collective invasion morphology was distinct from that observed with the SaGA-derived leaders (Figure 1, E and F). These findings build on the initial characterization in Figure 1, A–C, where we demonstrate that invasive heterogeneity and IL13RA2 heterogeneity are broadly observed across NSCLC cell lines and patient-derived samples. This context establishes that the molecular and phenotypic features explored in depth using the H1299 model system are not confined to a single cell line, but instead reflect a property of NSCLC heterogeneity. In the H1299 model system specifically, IL13RA2 can be utilized for the isolation of subpopulations with distinct invasive capacities.

### IL13RA2 and mitochondrial membrane potential characterize distinct subpopulations in NSCLC

**FIGURE 2:**
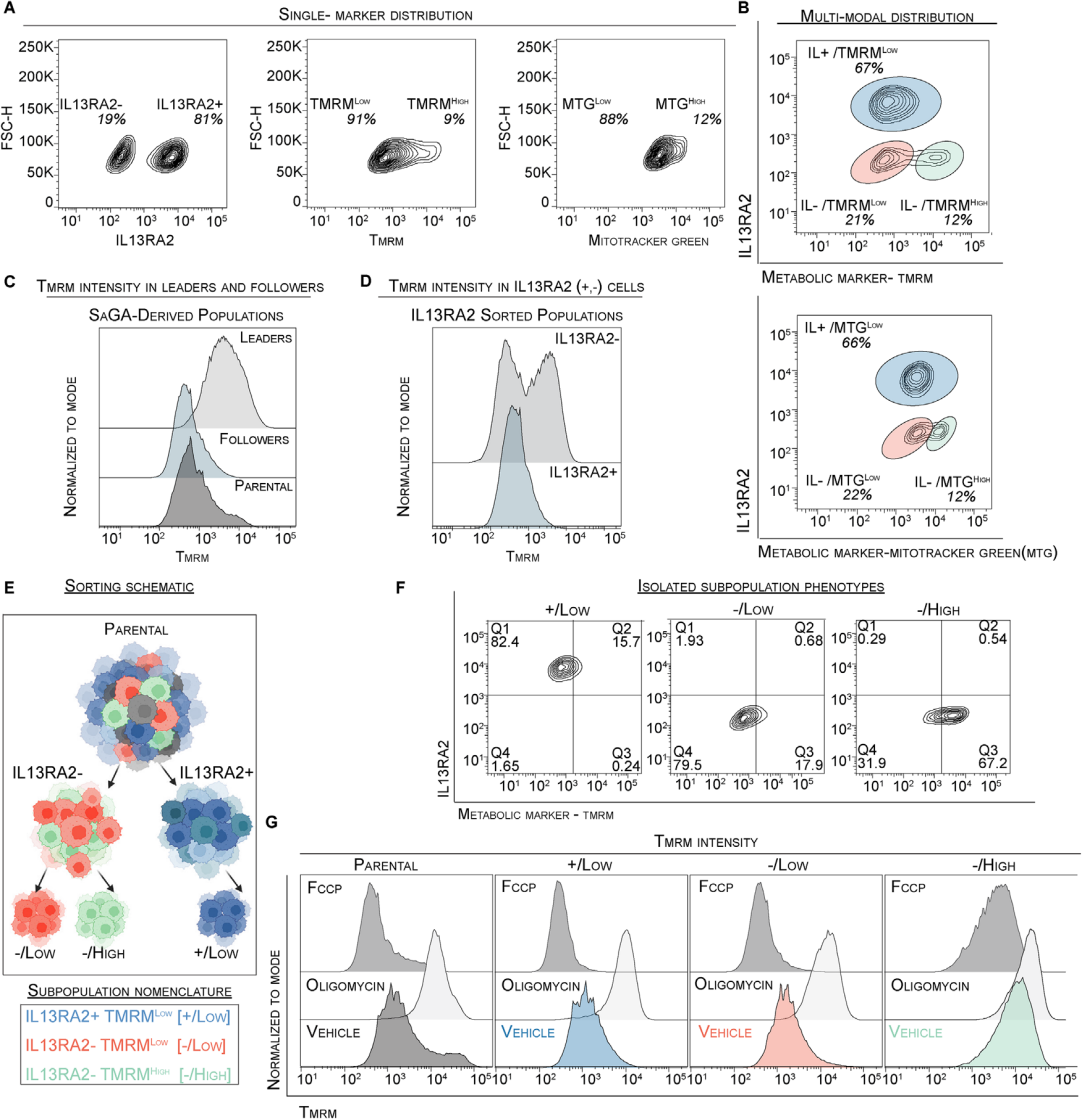
IL13RA2 and mitochondrial membrane potential characterize distinct subpopulations in NSCLC. (A) Flow cytometry quantification of IL13RA2, TMRM, and MitoTracker Green in H1299 parental cell line using live-cell flow cytometry (*n* = 3). (B) Live-cell flow cytometry of either IL13RA2 and TMRM or IL13RA2 and MitoTracker Green in H1299 parental cell line (*n* = 2). (C) Flow cytometry assessment of Parental, SaGA-derived Followers, SaGA-derived Leaders populations, TMRM intensity (*n* = 3). (D) Flow cytometry assessment of IL13RA2^+^ and IL13RA2^−^ populations, TMRM intensity (*n* = 3). Histograms normalized to the mode and representative of TMRM intensity are shown. (E) Schematic illustrating the sorting criteria used to identify and isolate +/Low, −/Low, and −/High cells via FACS. (F) Flow cytometry IL13RA2 and TMRM confirmation after subpopulation sorting (*n* = 3). (G) Flow cytometry detection of mitochondrial membrane potential using TMRM. FCCP or Oligomycin was added to samples incubated with TMRM, and then the cells were analyzed by flow cytometry (*n* = 2). Histograms normalized to the mode and representative of TMRM intensity are shown. See also Supplemental Figure S2.

Because leader cells demonstrate enhanced invasiveness and rely on mitochondrial respiration (Commander *et al.*, 2020), we investigated the heterogeneity of mitochondrial bioenergetics within our H1299 NSCLC model system, comparing our IL13RA2-sorted subpopulations with the SaGA-derived leader and follower subpopulations. During oxidative phosphorylation, cells generate a proton gradient between the intermembrane space and the matrix of the mitochondria, resulting in a mitochondrial membrane potential (ΔΨm) (Mitchell, 1966; Perry *et al.*, 2011; Zorova *et al.*, 2018). To assess ΔΨm, we used tetramethylrhodamine methyl ester (TMRM), a fluorescent cationic dye that accumulates in the mitochondrial matrix in proportion  to ΔΨm (Valdebenito and Duchen, 2022). The intensity of TMRM fluorescence correlates directly with ΔΨm, where a higher membrane potential leads to increased TMRM accumulation and fluorescent signal. Interestingly, although TMRM staining showed a biphasic distribution, the subpopulations identified via flow cytometry analysis of the ΔΨm revealed that the parental H1299 cell line contained ∼91% TMRM^LOW^ cells and 9% TMRM^HIGH^; this biphasic distribution (91:9) does not phenocopy the IL13RA2 distribution (70:30) (Figure 2A). Although TMRM corresponds to mitochondrial health and function based on membrane potential fluctuations, the net TMRM signal depends also on the mitochondrial volume within each cell (Distelmaier *et al.*, 2008). Therefore, we evaluated mitochondrial mass using MitoTracker Green FM, a cell-permeable fluorescent dye that labels active mitochondria in live cells independent of changes in mitochondrial membrane potential (ΔΨm) (Pendergrass *et al.*, 2004). Flow cytometry analysis of MitoTracker staining revealed that the parental cell line also composed of two distinct populations—MitoTracker^LOW^ cells and MitoTracker^HIGH^ cells—suggesting that TMRM intensity may parallel mitochondrial load (Figure 2A).

Since the biphasic distributions of our two approaches yielded distinct subpopulation percentages, we sought to integrate our two approaches for greater sensitivity. By combining, we combined our cell surface biomarker IL13RA2 with TMRM and identified three distinct subpopulations within the H1299 line (Figure 2B). Although flow cytometry analysis of IL13RA2 in conjunction with TMRM and/or MitoTracker confirmed a similar distribution for IL13RA2^+^ as observed when analyzing IL13RA2 alone, this approach allowed us to uncover previously undetected heterogeneity within our IL13RA2^−^ cells (Figure 2B). Our analysis showed that IL13RA2^−^ cells are composed of ∼21% TMRM^LOW^ (−/Low) cells and 12% TMRM^HIGH^ (−/High) cells (Figure 2B). Similar distributions were also observed when combining IL13RA2 with MitoTracker. To validate the TMRM phenotypes observed in the parental population in our IL13RA2-sorted and SaGA-derived subpopulations, we examined TMRM intensity within these isolated various subpopulations: SaGA-derived leaders, SaGA-derived followers, IL13RA2^+^ sorted cells, and IL13RA2^−^ sorted cells (Figure 2, C and D). Our analysis revealed similar TMRM intensities for the SaGA-derived followers (Figure 2C) and IL13RA2^+^ cells (Figure 2D), which make up ∼70% of the parental population, falling within the low ΔΨm population observed in the parental group. In contrast, the IL13RA2^−^ cells contained two distinct populations: one with high ΔΨm and one with a low ΔΨm (Figure 2D), similar to that observed with the SaGA-derived followers and IL13RA2^+^ cells. Interestingly, the SaGA-derived leaders (Figure 2C), which are IL13RA2^−^, showed a singular population with high ΔΨm, suggesting that the SaGA-derived leaders may reside within the IL13RA2^−^ ΔΨm-high subpopulation. These data establish a plausible technical approach for isolating metabolically distinct subpopulations previously undetected with a single-biomarker approach.

We proceeded to isolate three distinct subpopulations using differential IL13RA2 expression and ΔΨm: an IL13RA2^+^ subpopulation with low ΔΨm (+/Low), an IL13RA2^−^ subpopulation with low ΔΨm (−/Low), and an IL13RA2^−^ subpopulation with high ΔΨm (−/High) (Figure 2E). Once isolated from the heterogeneous parental populations, we confirmed that the three sorted subpopulations stably maintained their original IL13RA2 biomarker expression and ΔΨm status (Figure 2F). To verify ΔΨm status in relation to mitochondrial function, we used carbonyl cyanide-p-trifluoromethoxyphenylhydrazone (FCCP) and oligomycin. FCCP disrupts the proton gradient across the inner mitochondrial membrane, resulting in a reduction to complete collapse of ΔΨm (Terada, 1990). Conversely, oligomycin inhibits ATP synthase to increase ΔΨm. As expected, the addition of FCCP resulted in a decrease in TMRM fluorescence intensity, indicating mitochondrial depolarization (Figure 2G; Supplemental Figure S2A). Similarly, the introduction of oligomycin led to an increase in TMRM fluorescence intensity, signifying hyperpolarization of ΔΨm.

In parallel, we assessed whether MitoTracker Green was sensitive to changes in mitochondrial function and/or polarization state. Our three subpopulations (+/Low, −/Low, and −/High) maintained distinct mitochondrial loads (Supplemental Figure S2B). Given that MitoTracker Green exhibits low sensitivity to mitochondrial membrane potential (ΔΨm), we hypothesized that the introduction of FCCP and oligomycin would not significantly alter MitoTracker fluorescence. Our findings confirmed that the addition of FCCP did not reduce MitoTracker Green fluorescence; similarly, the introduction of oligomycin did not notably increase fluorescence levels (Supplemental Figure S2C). These data suggest that, while TMRM intensity is dependent on mitochondrial load in our model system, MitoTracker Green provides a ΔΨm - independent readout of mitochondrial load . Taken together, the isolated and stable +/Low, −/Low, and −/High subpopulations are products of a molecular isolation strategy designed to investigate and deconstruct metabolic heterogeneity.

### Stratification by IL13RA2 and mitochondrial membrane potential establishes phenotypically distinct subpopulations in NSCLC

**FIGURE 3:**
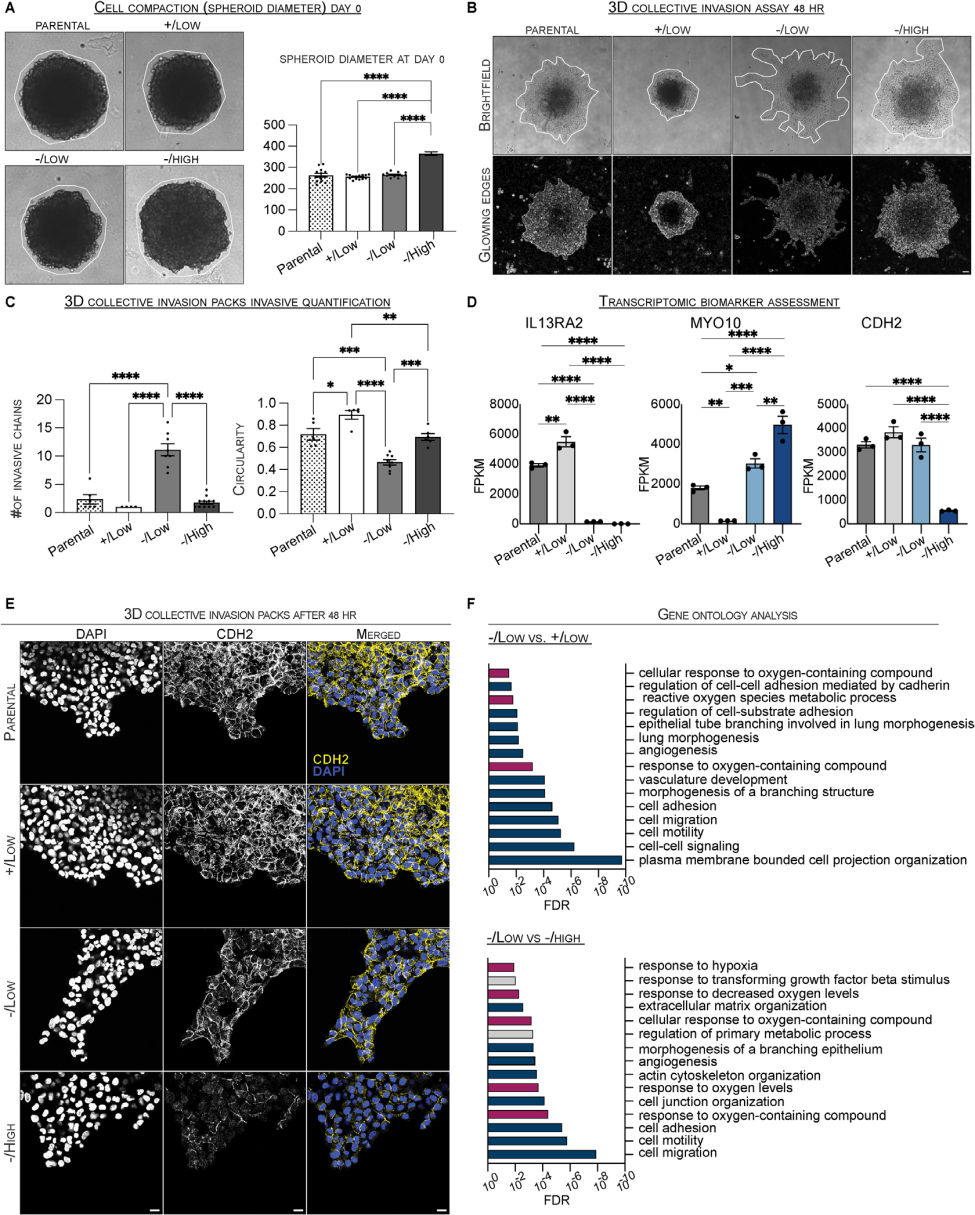
Stratification by IL13RA2 and mitochondrial membrane potential establishes phenotypically distinct subpopulations in NSCLC. (A) On day 0, cell spheroids of (parental, +/Low, −/Low, and −/High cells) were embedded in Matrigel, and cell compaction (spheroid diameter) was quantified (*n* = 3) and (*N* = 14 +/Low) (*N* = 13 −/Low) (*N* = 14 −/High) (*N* = 13 Parental). Representative brightfield images are shown. Solid (white) lines outline the outer perimeter of the spheroid; scale bar, 100 µm. (B) Spheroids of (parental, +/Low,−/Low, and −/High cells) were embedded into Matrigel and allowed to invade for 48 h. Representative images of cell invasion are shown. Solid (white) lines outline the outer perimeter of the invasive area after 48 h; scale bar, 100 µm. (*n* = 3) and (*N* = 5 +/Low) (*N* = 8 −/Low) (*N* = 12 −/High) (*N* = 6 Parental). (C) Quantification of the number of invasive chains and overall circularity of cells in the 3D collective invasion assay after 48 h. (*n* = 3) and (*N* = 5 +/Low) (*N* = 8 −/Low) (*N* = 12 −/High) (*N* = 6 Parental). (D) Bar graphs quantifying IL13RA2, MYO10, and CDH2 transcript levels in parental, +/Low, −/Low, and −/High populations. Three biological replicates were included in the analysis. Data are represented as mean ±SEM. (C and D) To assess significance, an ordinary one-way ANOVA was used with a Tukey's multiple comparisons test, ∗*p* ≤ 0.05, ∗∗*p* ≤ 0.01, ∗∗∗*p* ≤ 0.001, ∗∗∗∗*p* ≤ 0.0001. (E) 3D immunofluorescence imaging of parental, +/Low, −/Low, and −/High cell spheroids after 48 h with CDH2 marked in yellow and DAPI in blue; scale bar, 200 µm (*n* = 1). (F) PANTHER GO pathway analysis for the most distinct proteins. The *p*-value was <0.05, fold change >2, FDR <0.05. Dark blue indicates GO terms associated with migration/collective invasion. Pink indicates GO terms associated with oxygen/hypoxia. See also Supplemental Figure S3.

Given that collective invasion is driven by cooperative behaviors of distinct subpopulations (Shin *et al.*, 2014; Zhang *et al.*, 2019; Zoeller *et al.*, 2019; Commander *et al.,* 2020; Summerbell *et al.*, 2020; Hapach *et al.*, 2023; Zhang and Reinhart-King, 2023), we assessed how our isolated subpopulations form spheroids and invade within a 3D microenvironment. Notably, the −/High subpopulation had the largest spheroid diameter at day 0, suggesting an inability to aggregate into compact spheroid structures (Figure 3A). In contrast, parental, +/Low, and −/Low cells had similar spheroid diameters, suggesting differences in cell–cell adhesion compared with −/High cells. Next, we assessed the invasive behaviors (pack morphology, invasive area) of our isolated subpopulations after 48 h embedded in rBM (Figure 3B). Phenotypic analysis revealed that +/Low cells exhibited high contiguity and  circularity, lacking distinct invasive packs, consistent with SaGA-derived followers (Figure 3, B and C). Conversely, −/Low cells formed the largest number of invasive chains, consistent with SaGA-derived leaders (Figure 3, B and C). Intriguingly, −/High cells displayed an intermediate behavior in terms of invasiveness and pack morphology (Figure 3, B and C). −/High cells were neither as contiguous as +/Low cells, nor as invasive as −/Low cells, suggesting that −/High cells potentially reside within a middle ground between two distinct cellular phenotypes. These data suggest significant heterogeneity among these +/Low, −/Low, and −/High subpopulations. Given that −/High cells share mitochondrial membrane potential and IL13RA2 expression profiles with SaGA-derived leader cells, we expected them to exhibit similar invasive signatures. Conversely, we anticipated that +/Low cells would resemble SaGA-derived follower cells in their invasive phenotype. Although +/Low cells behaved as expected, the intermediate phenotype of −/High cells was unanticipated, suggesting that invasive phenotypes extend beyond a strict leader–follower dichotomy.

To determine how oxygen tension influences the underlying transcriptomes of heterogeneous subpopulations, we conducted RNA-sequencing (RNA-seq) on the H1299 parental cell line along with our subpopulations cultured under 21 and 1% O_2_ tension. Although the −/Low subpopulation displayed the most significant invasive phenotype after 1% O_2_ tension, we sought a comprehensive view of oxygen-dependent transcriptional programs. We therefore included all three subpopulations and parental cells to capture both shared/cooperative and distinct oxygen-related programs, without limiting analysis to invasion phenotypese. Principal component analysis (PCA) identified unique gene expression profiles for each group (Supplemental Figure S3A). To assess whether these subpopulations resembled SaGA-derived leaders and followers, we used a previously established biomarker-based approach (Zoeller *et al.*, 2019; Pedro *et al.*, 2020; Khatib *et al.*, 2025). Previously identified leader markers (MYO10, FN1, JAG1(Summerbell *et al.*, 2020), and CD70) were highly expressed in both −/Low and −/High cells (Figure 3D; Supplemental Figure S3B). SaGA-derived leaders are further characterized by the absence of IL13RA2 and low N-Cadherin expressions (Figure 3D), while being robustly expressed in SaGA-derived followers. CDH2(Cadherin-2) is a key cell–cell adhesion molecule involved in collective cell migration (Saénz-De-santa-maría *et al.*, 2020). Interestingly, although IL13RA2 expression was consistently low in both −/Low and −/High populations, CDH2 showed a variance between the subpopulations. CDH2 was highly expressed in +/Low and −/Low cells but minimally expressed in −/High cells. To investigate the differences in cell–cell adhesion properties, we performed 3D immunofluorescence analyses in the parental, +/Low, −/Low, and −/High cells. We observed robust N-Cadherin staining in the parental, +/Low, and −/Low invasion packs (Figure 3E). In contrast, although the −/High spheroids invaded collectively, the expression of N-Cadherin was significantly lower at the adherens junctions for the −/High cells, likely accounting for the less compact spheroid structure (Figure 3E). In summary, the three sorted populations demonstrate distinct gene expression patterns as well as distinct invasive morphologies.

To integrate transcriptomic data with functional phenotypes, we conducted Gene Ontology (GO) analysis across our subpopulations. Pairwise comparisons highlighted functional phenotypes implicated in collective invasion (migration, cooperation, and development), suggesting an underlying cooperative invasive potential of these subpopulations (Figure 3F; Supplemental Figure S3C). GO analysis emphasized migratory phenotypes via upregulation of cell migration, cell motility, cell adhesion, and ameboidal-type cell migration signatures. Cooperative phenotype signatures consisted of both cell–cell adhesion and cell–cell signaling, whereas developmental signatures included axonogenesis, angiogenesis, lung morphogenesis, vasculature development, tissue morphogenesis, and morphogenesis of a branching structure (Figure 3F; Supplemental Figure S3C). GO analysis also highlighted oxygen-responsive pathways, including decreased oxygen levels, and regulation of reactive oxygen species metabolic process, suggesting a differential sensitivity to oxygen levels (Figure 3F; Supplemental Figure S3C). These data demonstrate heterogeneity in 3D patterning, where these subpopulations invade with patterns ranging from high contiguity, high circularity invasion to discrete, highly structured chains and packs. This analysis revealed both shared hypoxia-responsive signatures and subpopulation-specific signatures, including differential regulation of hallmark pathways in hypoxia, glycolysis, oxidative phosphorylation, and invasion (Figure 3F; Supplemental Figure S3C). Collectively, these subpopulations provide a framework for assessing collective invasion behaviors under varying oxygen conditions. Together, these findings suggest that oxygen tension not only shapes invasive phenotypes but also captures distinct transcriptional states that support collective invasion.

### Modulating oxygen tension results in distinct transcriptional and phenotypic profiles

**FIGURE 4:**
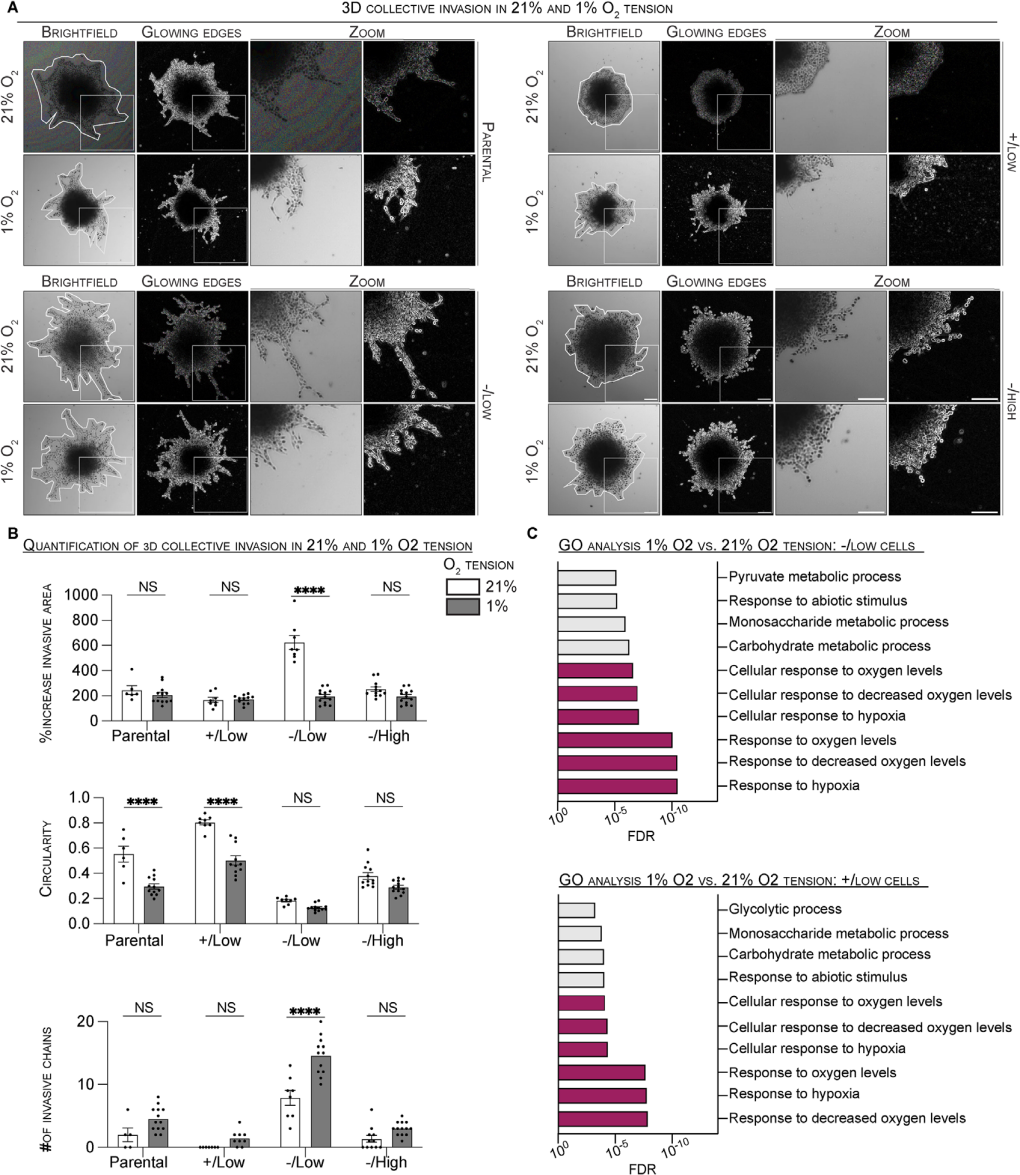
Modulating oxygen tension results in distinct transcriptional and phenotypic profiles. (A) Spheroids embedded in Matrigel were allowed to invade for 48 h either in 21% O_2_ tension or 1% O_2_ tension. Representative images of cell invasion are shown. Solid (white) lines outline the outer perimeter of the invasive area after 48 h; scale bar, 200 µm. (B) Quantification of invasive area and circularity after 48 h invasion in 21% O_2_ or 1% O_2_ tension. To assess significance, an ordinary one-way ANOVA with Tukey's multiple comparisons test was conducted, ∗*p* ≤ 0.05, ∗∗*p* ≤ 0.01, ∗∗∗*p* ≤ 0.001, ∗∗∗∗*p* ≤ 0.0001. In 21% O_2_ tension (*n* = 2) and (*N* = 8 +/Low) (*N* = 8 −/Low) (*N* = 12 −/High) (*N* = 6 Parental) and in 1% O_2_ tension –(*n* = 2) and (*N* = 11 +/Low) (*N* = 13 −/Low) (*N* = 13 −/High) (*N* = 12 Parental). (C) The PANTHER GO pathway analysis for the most distinct proteins after exposure to 1% O_2_ tension for pair-wise comparison between +/Low and −/Low cells. The *p*-value was <0.05, fold change >2, FDR <0.05. Pink indicates GO terms associated with oxygen/hypoxia. Three biological replicates were performed. See also Supplemental Figure S4.

Given that our sorted subpopulations display distinct and diverse metabolic phenotypes (mitochondrial load and polarization) and transcriptional signatures suggesting differential responses to oxygen levels, we explored how reducing oxygen tension from 21 to 1% O_2_ would impact 3D collective invasion. We hypothesized that lowering O_2_ tension would amplify behavioral differences among subpopulations with increased chain-like invasion as subpopulations escape toward adequate oxygenation. As such, we cultured spheroids in 21 or 1% O_2_ for 48 h and performed a phenotypic assessment (Figure 4A). Our results showed that 1% O_2_ reduced invasive circularity in all populations, with statistical differences observed in the parental and +/Low spheroids (Figure 4B). A decrease in circularity suggests an increase in chain-like,  and/or pack invasion patterns (Carey *et al.*, 2013; Konen *et al.*, 2017; Zoeller *et al.*, 2019; Summerbell *et al.*, 2020; Qin *et al.*, 2021; Zhang and Reinhart-King, 2023; Khatib *et al.*, 2025). Although there was an increase in the number of invasive chains across all subpopulations, there was only a significant increase in the −/Low subpopulation (Figure 4B). Notably, although the invasive areas of most subpopulations were largely unaffected by 1% O_2_ tension, −/Low cells showed a significant reduction in invasive area as the quantity of chains increased (Figure 4B). It is important to note that these comparisons reflect changes within each subpopulation across oxygen tensions (21 and 1% O_2_), rather than differences between subpopulations within a single oxygen condition. These findings partially aligned with our expectations—while all groups showed reduced circularity, only −/Low cells exhibited reduced invasive area, suggesting a unique sensitivity to the low-oxygen environment.

Next, we performed RNA-seq on cells cultured in 2D at 1% O_2_ tension for 48 h. GO analyses highlighted responses to low oxygen (response to decreased oxygen levels, oxygen levels, and hypoxia), suggesting that hypoxia is a significant regulator of cellular behavior in 2D cultures (Figure 4C; Supplemental Figure S4A). Additionally, GO analyses suggest metabolism also plays a critical role in low oxygen environments, with glycolytic, pyruvate, carbohydrate, and monosaccharide metabolic process signatures highlighted (Figure 4C; Supplemental Figure S4A). To further explore the biological processes relevant to cellular responses to hypoxia, we used the Molecular Signatures Database (MSigDB) to derive hallmark gene sets for hypoxia, glycolysis, oxidative phosphorylation, and invasion. Across these four hallmark gene sets, the individual subpopulations expressed unique and differential signatures, suggesting a cooperative manifestation of cellular behaviors in 21% (Supplemental Figure S4B) and 1% O_2_ (Supplemental Figure S4C) tension. Importantly, in 1% O_2_ tension, the −/High subpopulation exhibited a higher expression of genes across all four hallmark gene sets (compared with the +/Low and −/Low subpopulations), alluding to a strong biological response to low oxygen levels in 2D (Supplemental Figure S4C). Our transcriptomic analyses underscore the diverse behaviors present within heterogeneous populations cultured in both 3D (invasion assay) and 2D (transcriptomic). Taken together, these findings suggest that metabolism and hypoxia are major drivers of cellular behavior and may influence invasive behaviors in 3D environments. This supports our original hypothesis that oxygen levels not only impact individual subpopulation behavior but may also influence the dynamics of collective invasion.

### 3D invasive morphology in composite populations highlights additional subpopulations beyond the NSCLC leader–follower dynamic

**FIGURE 5:**
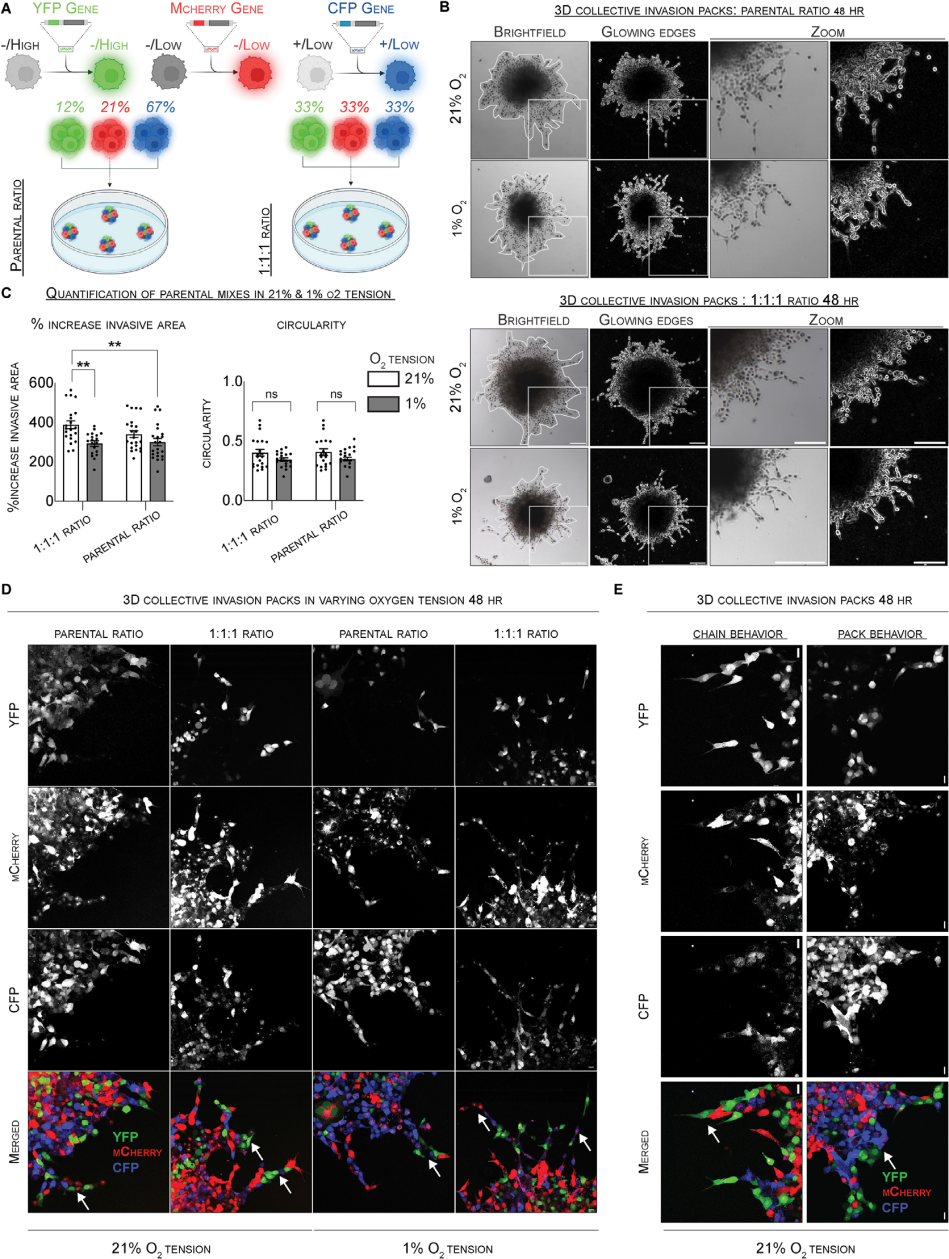
3D invasive morphology in composite populations highlights additional subpopulations beyond the NSCLC leader–follower dynamic. (A) Schematic detailing the experimental layout for the assessment of NSCLC patterning in +/Low, −/Low, and −/High cells. +/Low, −/Low, and −/High cells were transfected with YFP, mCherry, and CFP, respectively. The fluorescent populations were recombined in two distributions: the parental ratio and a 1:1:1 ratio, and then embedded in Matrigel and allowed to invade for 48 h. (B) Spheroids combined in the parental ratio or the 1:1:1 ratio were embedded in recombinant basement membrane matrix and allowed to invade for 48 h in either 21% O_2_ tension or 1% O_2_ tension. Representative images of cell invasion are shown. Solid (white) lines outline the outer perimeter of the invasive area after 48 h; scale bar, 200 µm. (C) Quantification of the percentage of increase in the invasive area and circularity. To assess significance, an ordinary two-way ANOVA with Tukey's multiple comparisons test was conducted, ∗*p* ≤ 0.05, ∗∗*p* ≤ 0.01, ∗∗∗*p* ≤ 0.001, ∗∗∗∗*p* ≤ 0.0001. (*n* = 4) and (*N* = 21 1:1:1 ratio 21% O_2_ tension) (*N* = 22 parental ratio 21% O2 tension) (*N* = 19 1:1:1 ratio 21% O_2_ tension) (*N* = 22 parental ratio 1% O_2_ tension). (D and E) Representative confocal images of 3D collective invasion in varying oxygen tensions with +/Low cells in CFP, −/Low cells in mCherry, and −/High cells in YFP. Merged images include YFP, mCherry, and CFP signals. Arrows indicate invasive projections extending from the spheroid into the surrounding matrix; scale bar, 100 µm.

To investigate how heterogeneous subpopulations collectively invade when recombined together, we stably transfected our three distinct subpopulations with constitutively expressed fluorophores: +/Low with CFP, −/Low with mCherry, and −/High with YFP (Figure 5A). With these labeled subpopulations, we evaluated how population composition impacts cooperative dynamics and collective invasion within a 3D microenvironment. We hypothesized that recombining the three subpopulations at their parental composition would recapitulate the invasive behavior of the H1299 parental line, whereas combining them at a 1:1:1 ratio might reveal cooperative or competitive dynamics not observed in isolation or in the bulk parental population. To this end, we recombined our three subpopulations into two defined distributions (Figure 5A). The first recapitulates the ratio of the subpopulations in the H1299 parental line, where ∼67% of the population is +/Low, ∼21% of the population is −/Low, and ∼12% of the population is −/High. The second used an equal 1:1:1 ratio of the subpopulations to create an equilibrium not typically observed in culture. After 48 h of 3D invasion, the spheroids were fixed and phenotypically analyzed (Figure 5B). There were no differences in invasive area or circularity between the two recombined populations in 21% O_2_ tension (Figure 5C). However, in 1% O_2_ tension, there was a significant decrease in invasive area in the 1:1:1 ratio group compared with 21%, which was not observed in the parental ratio (Figure 5C).

We next assessed collective invasion patterns in the two experimental groups (Figure 5D). In the parental ratio, +/Low cells (blue) were prominent within invasive projections. Interestingly, we also observed −/Low (red), and −/High (green) cells dispersed the SaGA-defined follower spatial positions. Across both experimental groups, −/Low and −/High cells traveled the greatest distances from the core of the spheroid, while +/Low cells predominantly occupied SaGA-derived follower-like spatial positions following behind. Oxygen levels were associated with distinct invasive patterning. In 21% O_2_ tension, invasive projections were broader and often exhibited chain-like organization. In contrast, in 1% O_2_ tension, invasion shifted toward thinner, tightly organized, single-file invasive projections. (Figure 5, B and D). In this low O_2_ tension environment, subpopulation patterning revealed −/Low and −/High cells localized to the tips of these invasive projections. In contrast, +/Low cells retained their follower-like spatial positions, distributing themselves throughout the invasive projections adjacent to −/Low and −/High cells.

Interestingly, our observations revealed invasive behaviors that extend beyond the typical leader–follower dynamics observed under 21% microenvironmental O_2_ (Konen *et al.*, 2017; Zoeller *et al.*, 2019; Commander *et al.*, 2020; Summerbell *et al.*, 2020; Khatib *et al.*, 2023; 2025). Although we observed single-file invasive chains with −/High in the leader-position and +/Low cells following behind, we also observed projections spanning multiple cells in width (Figure 5E). These invasive packs were spatially defined, suggesting organized pack formation. In some instances, a group of −/High cells was positioned at the front, while +/Low cells composed the rest of the invasive pack. In other cases, we found more complex spatial patterning of invasive packs with −/High cell at the front of the projection, followed by a −/Low cell; as the projection becomes composed of multiple cells in width, −/High cells and −/Low cells occupy the edges of the projection, with +/Low cells positioned in the center (Figure 5E). These data demonstrate that low O_2_ tension promotes a shift toward tightly organized, single-file invasive projections, while also revealing heterogeneous yet structured collective invasion patterning beyond the binary leader–follower dogma, providing biologically relevant tools to help dissect the underlying biology.

### 3D spatial positioning within composite populations is opportunistic and dependent on oxygen availability

**FIGURE 6:**
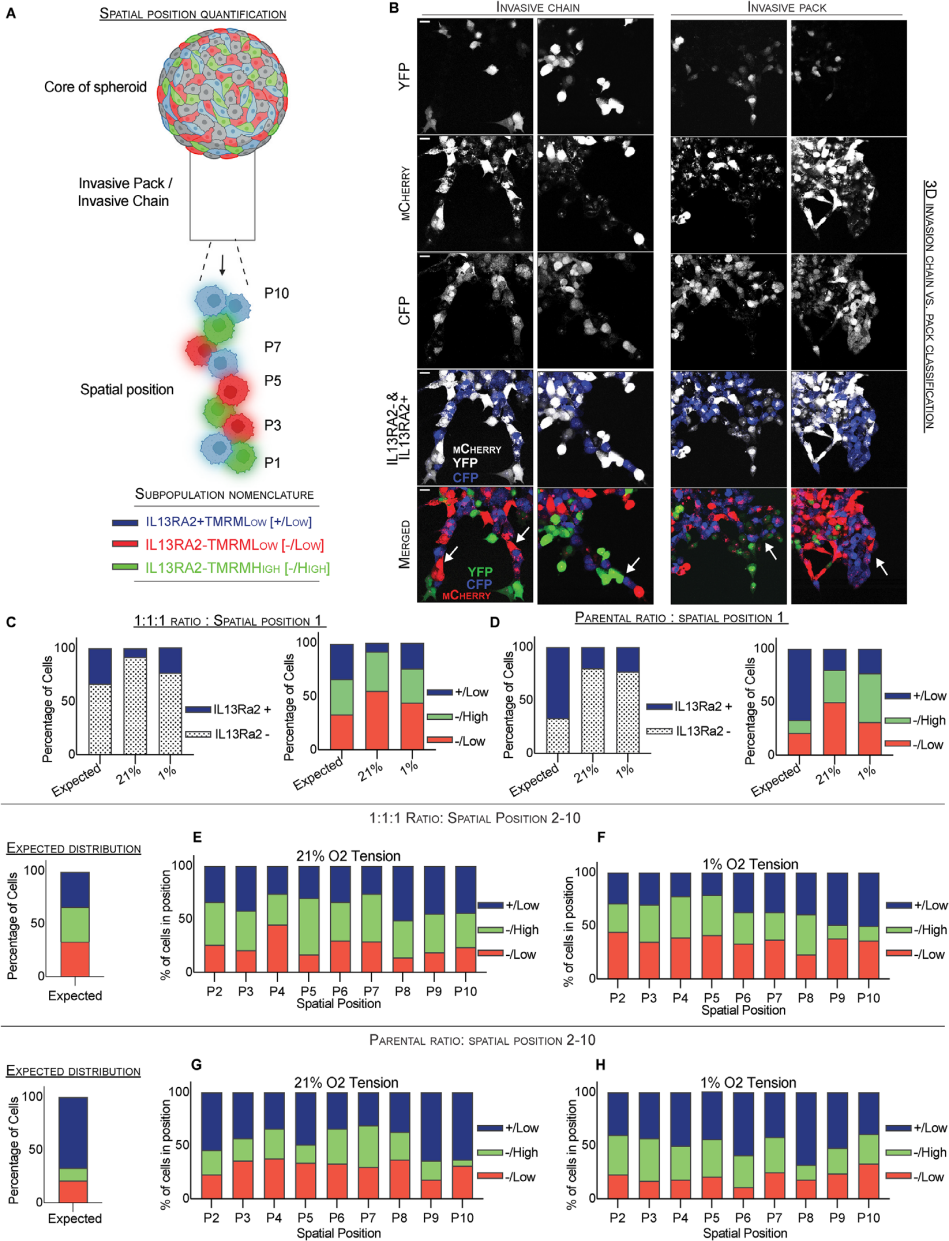
3D spatial positioning within composite populations is opportunistic and dependent on oxygen availability. (A) Schematic detailing the spatial position quantification of invasive packs/chains emerging from the core of the spheroid. (B) Representative confocal images of 3D collective invasion with +/Low cells in CFP, −/Low cells in mCherry, and −/High cells in YFP. Merged images include mCherry and YFP in white with CFP in blue. Merged images also include YFP, mCherry, and CFP signals; scale bar, 100 µm. (C) Bar graphs quantifying the distribution of IL13RA2^−^ (−/Low, −/High) and IL13RA2^+^ cells in spatial position 1 in spheroids composed of a 1:1:1 ratio of +/Low (blue), −/Low (red), and −/High (green) cells. 1:1:1 ratio in 21% O_2_ tension (*n* = 3) and (*N* = 46). 1:1:1 ratio in 1% O2 tension (*n* = 3) and (*N* = 42). (D) Bar graphs quantifying the distribution of IL13RA2^−^ (−/Low, −/High) and IL13RA2^+^ cells in spatial position 1 in spheroids composed of a parental ratio of +/Low (blue), −/Low (red), and −/High (green) cells. Parental ratio in 21% O2 tension (*n* = 3) and (*N* = 39). Parental ratio in 1% O_2_ tension (*n* = 3) and (*N* = 43). (E) Bar graphs quantifying the distribution of +/Low, −/Low, and −/High cells in spatial position 2 through 10, in spheroids composed of 1:1:1 ratio of +/Low, −/Low, and −/High cells, in 21% O_2_ tension. (F) Bar graphs as in E, but in 1% O_2_ tension. (G) Bar graphs quantifying the distribution of +/Low, −/Low, and −/High cells in spatial position 2 through 10, in spheroids composed of a parental ratio of +/Low, −/Low, and −/High cells, in 21% O_2_ tension. (H) Bar graphs as in G, but in 1% O_2_ tension. For all experiments, three biological replicates were performed, with at least 40 invasive projections analyzed for each treatment. See also Supplemental Figure S6 and Supplemental Tables S1 to S4 for statistical analysis.

To investigate how subpopulation heterogeneity integrates with varying oxygen tensions during collective invasion patterning, we developed a methodology for spatial position quantification. Spheroids containing three fluorescent populations—+/Low (blue cells), −/Low (red cells), −/High (green cells)—were combined  at two recombination ratios—1:1:1 or the observed parental composition—and embedded into rBM (Figure 5A). After 48 h of invasion, the spheroids were fixed and phenotypically analyzed. Using confocal imaging, we assigned the first cell in each collective unit as position 1, modeled after leader spatial positioning (Konen *et al.*, 2017; Zoeller *et al.*, 2019; Commander *et al.*, 2020; Summerbell *et al.*, 2020; Khatib *et al.*, 2023). As we moved from this position 1 toward the core of the spheroid, we incremented the position number, arbitrarily stopping the count at position 10 (Figure 6A). During analysis, we defined “packs” as cohesive groups of cells that move together as a unit, while “chains” were identified as linear arrangements where cells moved in single-file formations (Figure 6B).

To assess leader spatial patterning in 21% O_2_ tension, we analyzed the distribution of cells at position 1 in both the 1:1:1 and parental ratios. In the 1:1:1 ratio, while the predicted composition of position 1 was 66% IL13RA2^−^ cells (−/Low and −/High combined), the observed composition was ∼90% (Figure 6C). Expected values for each ratio were calculated based on the initial recombination ratios of the mixed spheroids (67% +/Low:21% −/Low:12% −/High, 1:1:1 ratio). Deviations from expected values were tested using *χ*^2^ analyses. In the parental ratio, the expected composition of IL13RA2^−^ cells at position 1 was 33%, and the observed composition at position 1 was ∼80%, comprising  either −/Low or −/High cells (Figure 6D). Further analysis revealed the percentages of −/Low cells were significantly above the expected values in both ratios, supporting enrichment of −/Low cells at position 1 (Figure 6, C and D; Supplemental Figure S6A; Supplemental Tables S1 and S3). +/Low cells were observed at 25 and 47%, in contrast with their expected 33% and 67%, respectively, suggesting an inhibition of +/Low cells at position 1 (Figure 6, C and D; Supplemental Figure S6A). Interestingly, in the 1:1:1 ratio, −/High cells were observed at approximately their expected values; however, in the parental ratio, −/High cells were significantly enriched at position 1 (Figure 6, C and D; Supplemental Figure S6A; Supplemental Tables S1 and S3). Taken together, these data suggest that position 1 is characterized by the presence of IL13RA2^−^ cells with −/Low cells maintaining a leader spatial phenotype in 21% O_2_ tension (Supplemental Tables S1 and S3). Although −/High cells are more equipped to reach position 1 than +/Low cells, they are not as efficient as −/Low cells (Supplemental Tables S1 and S3). The *χ*^2^ analyses confirmed that the observed distributions significantly differed from expected values (*p*<0.05), demonstrating that leader positions are not occupied stochastically but are enriched for specific subpopulations (Supplemental Tables S1 and S3).

To further assess the spatial patterning within the collective invasion unit, we continued to increment phenotypic assessments from position 2 to 10. Our initial hypothesis was that +/Low cells would maintain the largest percentage from position 2 to 10, recapitulating follower-invasive behaviors. However, our results indicated a more complex behavior (Supplemental Figure S6B). In spheroids composed of either a 1:1:1 ratio or a parental ratio, we observed IL13RA2^−^ cells displayed follower-like spatial patterning, as they were found throughout the invasive projection trailing behind position 1 (Figure 6, E and G; Supplemental Figure S6C). Upon further inspection, we found spatial patterning was distinct in both ratios of the subpopulations, alluding to inherent differences in collective behaviors. Initially, we found that the 1:1:1 ratio maintained expected percentages across all three subpopulations, with notable increases in +/Low cells from position 8-10 and −/High cells at positions 5 and 7 (Figure 6E; Supplemental Figure S6, C and D). The percentage of −/Low cells observed fluctuated significantly from the predicted, alternating between being below and above expected levels throughout the invasive projection. Overall, the data from the 1:1:1 ratio suggest a stochastic subpopulation localization throughout the collective invasion unit while highlighting a substantial presence of IL13RA2^−^ cells. In contrast, the quantification of the spatial patterning observed with the parental ratio described a non-stochastic underlying driver for spatial patterning. Notably, +/Low cells exhibited spatial patterning similar to the predicted, trailing behind leading cells (Figure 6G; Supplemental Figure S6C). The percentage of +/Low cells steadily increased beyond position 1, rarely falling below 40% at positions 2-10. Despite expecting only ∼10% (−/High) and ∼20% (−/Low) cells, both were found in significantly higher numbers than expected in positions 2 to 10. This suggests that their invasive potential is not limited to leading the invasive projection and therefore they play a broader role in the process.

Given the differential sensitivity observed in the subpopulations in 1% O_2_ conditions, we investigated how low oxygen levels impact collective invasion within a heterogeneous population.

Spheroids recombined in both the 1:1:1 and parental ratios were incubated in 1% O_2_ tension for 48 h and then fixed for phenotypic analysis. IL13RA2^−^ cells maintained their significant presence at position 1 (Figure 6, C and D). However, distinct spatial localization patterns emerged in low oxygen. In the 1:1:1 ratio, the percentage of cells at position 1 was closer to expected levels compared with 21% O_2_ tension, alluding to less phenotypically determined spatial positions in low oxygen (Figure 6C; Supplemental Figure S6A; Supplemental Table S2). In contrast, in the parental ratio, we observed fewer −/Low cells at position 1 compared with 21% and a higher percentage of −/High cells (Figure 6D; Supplemental Figure S6A; Supplemental Table S4). These data suggest that −/High cells are suited to lead the invasive front in the parental distribution of subpopulations under low-oxygen conditions. To determine whether spatial patterning is maintained in 1% O_2_ tension, we continued to increment phenotypic assessments from position 2-10 in both recombination ratios (1:1:1 versus parental) of the subpopulations. In the 1:1:1 ratio, IL13RA2^−^ cells generally maintained their expected distribution, with the exception of positions 9 and 10, where there was a notable increase in +/Low cells (Figure 6F; Supplemental Figure S6C; Supplemental Table S2). In contrast, in the parental ratio, as we continued to increment from position 2 to 10, we observed a gradual increase in +/Low cells, often exceeding the distribution of IL13RA2^−^ cells (Figure 6H; Supplemental Figure S6C; Supplemental Table S4). Interestingly, −/High cells were consistently found in higher numbers than expected throughout the invasive projection (Figure 6H; Supplemental Figure S6D; Supplemental Table S4), maintaining levels greater than −/Low cells, indicating differential invasive capabilities under 1% O_2_ tension in a heterogeneous group. The *χ*^2^ analyses confirmed that these deviations were statistically significant (p<0.05), reinforcing that positional hierarchies are phenotype and oxygen-dependent rather than stochastic (Supplemental Tables S1–S4). Taken together, these data support a relationship between population distributions and spatial patterning in varying oxygen tensions.

## DISCUSSION

Intratumoral heterogeneity drives collective invasion (Zhang *et al.*, 2019; Zoeller *et al.*, 2019; Commander *et al.*, 2020; Summerbell *et al.*, 2020; Zhang and Reinhart-King, 2023; Sharma *et al.*, 2024; Khatib *et al.*, 2025), but whether phenotypic heterogeneity in NSCLC exists beyond a binary leader–follower dynamic, and how these dynamics are influenced by the population composition and microenvironment, remain largely unexplored. In this study, we leveraged molecular and metabolic findings to investigate whether heterogeneous subpopulations cooperate to support collective invasion. Our approach combined in vitro 2D and 3D methods to assess both isolated subpopulations and heterogeneous recombined subpopulations. Our results demonstrate dynamic cellular behaviors that facilitate differential collective invasion patterning with varying composite populations and oxygen tension.

Our in vitro studies in both 2D and 3D support the existence of phenotypic heterogeneity at multiple levels. On a population level, we identified distinct invasion patterns (noninvasive, single-cell invasion, highly contiguous, single-file chain-like) across multiple NSCLC cell lines and patient-derived samples (Figure 1, A and B). At the molecular level, these NSCLC cell lines and patient-derived samples contain subpopulations that exhibit heterogeneous expression of the cell surface marker IL13RA2 (Figure 1C). On a subpopulation level, IL13RA2^−^ cells are more invasive than their IL13RA2+ counterparts, consistent with our previous findings (Summerbell *et al.*, 2020) (Figure 1, E and F). However, IL13RA2 sorted populations fail to establish distinct invasion patterns beyond structured invasive chains (−/Low and SaGA-derived leaders) and highly contiguous invasion (+/Low and SaGA-derived followers) observed with our SaGA-derived populations (Konen *et al.*, 2017; Khatib *et al.*, 2025).

Our previous studies have revealed a gradient of glucose uptake in cells trailing leader cells, suggesting collective invasion patterning may be driven by metabolic phenotypes (Commander *et al.*, 2020). As such, we established a method for incorporating metabolic profiling in the assessment of heterogeneity. Combining the molecular marker IL13RA2(±) and the mitochondrial polarity proxy TMRM (Low/High), we obtained sufficient resolution to isolate three distinct subpopulations based on their IL13RA2 status and mitochondrial membrane potential (+/Low, −/Low, and −/High). We found that, on a subpopulation level, these subpopulations display heterogeneous metabolic (Figure 2G; Supplemental Figure S2C), transcriptional (Figure 3D; Supplemental Figure S3B), and invasive signatures (Figure 3, B and C). In 2D, the −/High subpopulation shares metabolic phenotypes (mitochondrial polarization/load) with SaGA-derived leaders but lacks their invasive capacity (structured cohesive chains). Whereas the −/Low subpopulation lacks the mitochondrial signatures of SaGA-derived leaders but are significantly more invasive than the +/Low and −/High cells. Differential regulation of key invasive genes implicated in collective invasion (JAG1, FN1, and CDH2), along with extensive GO functional phenotypes related to migration, cooperation, and development, suggests that invasive signatures extend beyond 3D behaviors (Figure 3).

We propose that an underlying cooperative invasive phenotype exists across these subpopulations, indicating that their interactions involve more complex behaviors than simply leading and following in contiguous chains. Although our findings are snapshots in time, subpopulations likely communicate and cooperate in real-time (Matise *et al.*, 2012; Zhang *et al.*, 2019; Zoeller *et al.*, 2019; Commander *et al.*, 2020; Summerbell *et al.*, 2020; Hapach *et al.*, 2023; Zhang and Reinhart-King, 2023; Khatib *et al.*, 2025). One avenue of cooperation is mediated by follower cells secreting TGFB1, which enhances cellular invasion and promotes leader cell proliferation for survival and colonization in stress-inducing secondary sites (Khatib *et al.*, 2025). Other factors mediating communication remain unknown, but our data suggest that subpopulations unable to invade in hypoxia alone likely benefit from cooperation at both primary and secondary sites. The −/Low cells are less invasive in low oxygen conditions (Figure 4B) but, upon adequate oxygenation, pave the way for +/Low and −/High cells via contiguous pack formation (Figure 6). It is possible that +/Low cells do not lead the invasive projection because they are glycolytically capable, allowing them to survive without adequate oxygenation and promote pack formation and survival of −/Low and −/High subpopulations as they invade and reach secondary tissues. Ultimately, invasive patterning can be a product of both the need for nutrients (Warburg *et al.*, 1927; Dang and Semenza, 1999; Heiden *et al.*, 2009), like oxygen (Helmlinger *et al.*, 1997; Gatenby and Gillies, 2004; Le *et al.*, 2006), and the capacity to opportunistically seek them. In 1% O_2_ conditions, −/High cells are enriched in the invasive projections. This is potentially because they are uniquely dependent on oxygen for survival, making them a strong sensor of oxygen levels. As such, they may effectively pilot the way for other cell populations to areas with sufficient oxygen. Whether all three subpopulations are required for the metastatic cascade remains unknown. However, MDA-MB-231 subpopulations dynamically rearrange leader and follower positions in response to the energetic requirements of invasion, which suggests all three populations might indeed play unique collaborative roles over the course of collective invasion and tumor progression (Zhang *et al.*, 2019). As this approach is a snapshot after 48 h of invasion, the requirements at the early timepoints of collective invasion are potentially unlike those observed for the duration of the 48 h, suggesting a need for spatiotemporal assessments of patterning.

Collectively, this work supports a model whereby collective invasion patterning is a product of both cell-intrinsic and cell-extrinsic factors. In a highly invasive NSCLC model, we identified three subpopulations with metabolic, transcriptional, and invasive heterogeneity, all driven by a common goal of invasion and survival. Our work contributes to a more complete understanding of how metabolic and invasive heterogeneity shape collective invasion patterning among heterogeneous subpopulations, which we anticipate will be critical in shaping anti-invasion therapies for heterogeneous diseases.

## MATERIALS AND METHODS

Request a protocol through *Bio-protocol*

### Experimental model subject details

#### Cell lines

NSCLC cell lines H1299, H23, H1915, H1975, H1755, H2228, H460, H549, and SKMES1 were purchased from the ATCC (Manassas, VA) and cultured in RPMI-1640 media supplemented with 10% FBS and 1% penicillin–streptomycin (P/S) and incubated at 37°C and 5% CO_2_. Hypoxia experiments were cultured at 37°C, 5% CO_2_, and 1% O_2_. NSCLC patient-derived cell lines CK10151, CK9121, and CK5494 were procured from the Patient-Derived Models Repository of the NIH NCI Division of Cancer Treatment and Diagnosis and grown in M87 media containing 2% FBS (DeRose *et al.*, 2013). Leader and follower subpopulations were derived from the H1299 cell line and isolated via the SaGA method as described previously (Konen *et al.*, 2017). Cell lines were passaged at 70 to 80% confluency, and both adherent and nonadherent cells were collected at each passage by collecting the media at the time of passage. Media were replaced every 2 d, and nonadherent cells were spun down. All cell lines tested negative for *Mycoplasma* contamination using a commercially available kit (PCR-*Mycoplasma* Test Kit I/C, Promokine PK-CA91-1024) before use in experiments.

### Method details

#### 3D Spheroid invasion assays

3D invasion assay was performed as described previously (Konen *et al.*, 2017). A total of 3000 cells/well were seeded in low-attachment 96-well plates (Corning) and incubated for 2 d to form spheroids. Spheroids were then embedded in 2:1 recombinant basement membrane (rBM, Matrigel, Corning) and modified culture media containing 5% FBS and 1% P/S. At a minimum, four spheroids were embedded per treatment, per biological replicate. Spheroids were allowed to invade for 48 h. Using an Olympus CKX41 microscope with Infinity 1-3C camera (x4 air, 0.13 numerical aperture (NA), UPlanFL N), we captured images at days 0 and 2 of invasion. Invasive surface area, spheroid circularity, and number of chains were quantified using FIJI, as described previously (Khatib *et al.*, 2023). In brief, the invasive area was quantified by outlining the perimeter of the spheroids using FIJI (Schindelin *et al.*, 2012) and calculated by subtracting the day 0 spheroid area from the day 2 area. Diameter was measured by measuring length across the spheroid using FIJI.

#### Western blot

For protein level analysis, 1.5 × 10^5^ cells were seeded in 10-cm plates and grown for 24 h in complete media. The media were changed the following day to treatment media containing 5% FBS and 1% P/S. After 48 h, cells were rinsed once with PBS and lysed with lysis buffer (2% SDS, 50 mM Tris pH 8.0, 100 mM NaCl, 1% Halt protease/phosphatase inhibitors; Thermo Fisher Science, 78442). Lysed samples were scraped, collected, and briefly sonicated to shear the DNA. Protein concentration was determined by Pierce BCA assay (Thermo Fisher Science, 23227). Proteins were separated using SDS–PAGE, transferred to nitrocellulose membranes, and detected using the Bio-Rad ChemiDoc Imaging System (Bio-Rad, 12003153).

##### Flow cytometry analyses

A total of 8 × 10^5^ cells were seeded in 10-cm plates and grown for 24 h. The next day, attached and floating cells were collected, pelleted at 200 × *g* for 5 min, washed with PBS, pelleted at 200 × *g* for 5 min, and resuspended in flow buffer (RPMI, no phenol red) with or without CD213α2 (IL13Rα2) antibody (Miltenyi Biotec, 130-128-220). Cells were incubated for 30 min at 4°C with gentle agitation in the dark. Cells were then washed twice, resuspended in flow buffer, and analyzed. For all experiments, flow cytometry analysis was performed using the BD FACSYMPHONY A3 system with the BD FACSDiva software. The following analysis and quantification were performed using FlowJo.

##### IL13RA2 flow cytometry sorting

H1299 cells (2 × 10^6^) were seeded in 15-cm plates and grown for 24 h. Attached and floating cells were collected the following day as a pellet, washed, and resuspended in flow buffer (RPMI, no phenol red) with or without IL13RA2 antibody (Militenyl Biotec,130-128-220). Cells were incubated for 30 min at 4°C with agitation in the dark. Cells were then washed 2x and pelleted and resuspended in flow buffer for sorting. Cell sorting was performed with a FACS Area IIB instrument (BD Biosciences). IL13RA2^+^ and IL13RA2^−^ populations were defined based on bimodal fluorescence intensity distributions, with one peak corresponding to low/negative fluorescence (IL13RA2^−^) and the second peak to higher fluorescence (IL13RA2^+^). Gates were drawn around these peaks for cell sorting.

##### Mitochondrial flow cytometry assays

Using tetramethylrhodamine methyl ester (TMRM, Thermo Fisher Scientific, I34361) mitochondrial membrane potential (Δ*Ψ)* was measured. A total of 9 × 10^5 ^cells were seeded in 10-cm plates and grown for 24 h. Attached and floating cells were collected the following day as a pellet, washed, and resuspended in flow buffer (RPMI, no phenol red) with or without 2 nM TMRM. Cells were incubated for 30 min at 37°C with light agitation, washed 2x, pelleted, and resuspended for analysis via flow cytometry using BC FACSymphony A3. TMRM-High and TMRM-Low populations were defined based on relative fluorescence intensity peaks within the parental H1299 population. A smaller peak (∼10^4^) was gated as TMRM-High, while the larger peak just below (∼10^3^) was gated as TMRM-Low. Pharmacological controls (FCCP and Oligomycin) validated these gates: FCCP collapsed mitochondrial membrane potential, shifting cells into the low gate, while Oligomycin increased mitochondrial membrane potential, shifting cells into the high gate.

Mitochondrial load was detected and quantified using 20 nM MitoTracker Green (Thermo Fisher Scientific/Invitrogen, M7514). Cells were incubated for 30 min at 37°C with light agitation. Samples were then washed, pelleted, and resuspended for analysis via flow cytometry on BC FACSymphony A3. MitoTracker^HIGH^ and MitoTracker^LOW^ populations were defined by distinct peaks in the parental population histogram, with gates set between peaks. For experiments using carbonyl cyanide-p-trifluoromethoxyphenylhydrazone (FCCP) and oligomycin, cells were prepared as described above. During the incubation with TMRM or MitoTracker Green, 200 nM FCCP (MilliporeSigma, C2920) or 1 µM oligomycin (MilliporeSigma, 495455) was added to the cells. Samples were then processed as above.

##### Mitochondrial membrane potential sorting

H1299 IL13RA2^−^ and IL13RA2^+^ sorted cells were plated at 2 × 10^6 ^cells per 15 cm plate and grown for 24 h. Both attached and floating cells were collected the following day as a pellet, washed, and resuspended in flow buffer (RPMI, no phenol red) with or without 2 nM TMRM. Cells were incubated for 30 min at 37°C with light agitation, washed 2x, pelleted, and resuspended in flow buffer for sorting. Cell sorting was performed with a FACS Area IIB instrument (BD Biosciences). For sorting of cells based on ΔΨm, IL13RA2^−^-gated cells were sorted into two populations corresponding to the cells with the lowest TMRM fluorescence and the highest TMRM fluorescence. IL13RA2^+^-gated cells were sorted based on the lowest TMRM fluorescence. Within the IL13RA2^−^ population, cells with the lowest TMRM fluorescence intensity were sorted as TMRM-Low, while those with the highest fluorescence were sorted as TMRM-High.

#### RNA-Seq

##### Sequencing, alignment, and quantification

Emory EPC Genomics Core checked sequences for quality using FastQC for completeness, depth, and overall read quality. To remove adapter contamination, data were trimmed using Trimmomatic (Bolger *et al.*, 2014). Trimmed sequences were aligned to the human reference genome (hg38) using the STAR aligner (Dobin *et al.*, 2013). Gene quantification was performed using HTSeq-count (Anders *et al.*, 2015).

#### Differential expression

Using DESeq2, differential gene expression analysis was performed. DESeq2 compares gene expression between at least two experimental groups (Love *et al.*, 2014). DESeq2 assumes that the gene expression count table follows a negative binomial distribution and uses the Wald test for statistical analysis. Genes with low counts are filtered using mean-normalized counts. Raw *p* values are adjusted for multiple hypothesis testing using the Benjamini–Hochberg correction. Genes with adjusted *p* values, also known as false discovery rates (or FDR), below the predetermined threshold (0.05) are considered significantly differentially expressed. Visualization plots are generated using specific package-recommended data transformations.

#### Cell lines and transfections

eYFP (GeneCopoeia, LP401-025), mCherry (GeneCopoeia, LP441-025), and eCFP (GeneCopoeia, LP421-025) lentiviral particles were purchased from GeneCopoeia and stored at -80°C until use. A total of 5 × 10^4^ cells/well were plated into 6-well plates in complete media. After 24 h, −/High cells were transduced with eYFP viral particles, +/Low cells were transduced with eCFP viral particles, and −/Low cells were transduced with mCherry viral particles. After 48 h, 1 µg/mL puromycin was added to the complete growth media per well. When drug-resistant colonies became visible, puromycin was removed.

#### Spheroid microscopy

##### Experimental set-up

A total of 3 × 10^3^ cells/well, composed of 33% +/Low, 33% −/Low, and 33% −/High (termed 1:1:1 ratio) were seeded in low-attachment 96-well plates and incubated for 3 d to form spheroids. Embedding and processing were performed as described previously. Spheroids were incubated for 48 h, fixed, and analyzed. A total of 3 × 10^3^ cells/well, composed of 67% +/Low, 21% −/Low, and 12% −/High (termed parental ratio), were seeded in low-attachment 96-well plates and incubated for 3d to form spheroids. Embedding and processing were performed as described previously. Spheroids were fixed after 48 h of 3D invasion with prewarmed 4% paraformaldehyde (PFA) for 40 min at room temperature with light agitation, and then followed by three 5 min washes with PBS/glycine (100 mM glycine).

##### Fixed cell confocal

Spheroids were imaged using the Leica TCS SP8 inverted confocal microscope (20X air HC PL APO CS2, 0.75 NA) using z-stack intervals, 4x line averaging, and sequential scanning (405, 514, and 561 nm).

##### Spheroid image analysis

FIJI (ImageJ) was utilized for image analysis and quantification. For each recombination ratio, ≥15 images swere taken per treatment and quantified. Positional counts of cells were incremented from the furthest point of the invasive projection (position 1) toward the core of the spheroid, arbitrarily ending at the 10th cell. Chain and pack length were noted for further quantification.

To determine whether specific subpopulations were enriched in positions, we compared observed positional frequencies with expected frequencies derived from the initial recombination ratios of the mixed cultures (67% +/Low, 21% −/Low, 12% −/High, or 1:1:1). These expected values represent the null hypothesis where cells distribute randomly, independent of molecular and invasive phenotype. Deviations from the expected values were assessed using *χ*^2^ analyses, with *p* values, indicating whether observed ratios differed from random dispersal throughout the invasive projection.

### Quantification and statistical analysis

Following validation of normal distribution, uniform variance, and independent sampling, the statistical tests specified in the figure legends were performed. Biological replicates are denoted as “*n*” and technical replicates as “*N*.” All *p* values were two-tailed. Microsoft Excel, ImageJ, and GraphPad Prism v10.2.3 were used for statistical analyses and the generation of graphs. Data are represented as mean ± SEM. *p ≤ 0.05, **p ≤ 0.01, ***p ≤ 0.001, ****p ≤ 0.0001. 

## Supporting information





## Abbreviations used:

−/High = IL13RA2 negative: TMRM high −/Low = IL13RA2 negative, TMRM low +/Low= IL13RA2 positive, TMRM low
FCCP: carbonyl cyanide-p-trifluoromethoxyphenylhydrazone
GSEA: gene set enrichment analysis
IL13RA2: interleukin 13 receptor alpha 2
MSigDB: Molecular Signatures Database
NSCLC: nonsmall cell lung cancer
rBM: recombinant basement membrane
SaGA: Spatiotemporal Genomic Analysis
TMRM: tetramethylrhodamine methyl ester (mitochondrial membrane potential probe).
