# Supplementary material for: Metabolic programming defines oxygen-sensitive subpopulation hierarchies and patterning in collective invasion
Source: Mol Biol Cell. 2025 Oct 16;36(11):ar137. doi: 10.1091/mbc.E25-07-0314 (PMC12562065; doi:10.1091/mbc.E25-07-0314)
Supplement: Supplementary file 1 [file mbc-36-ar137-s001.pdf]

Supplemental Materials

Molecular Biology of the Cell

Matsuk *et al*.

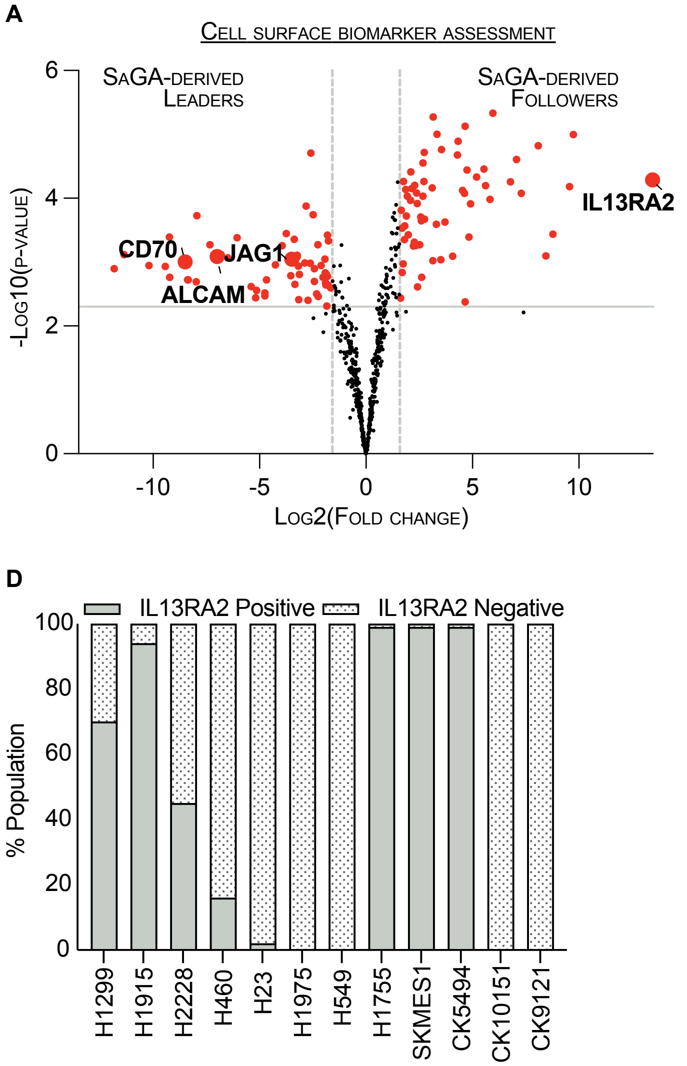

A
Cell surface biomarker assessment
SaGA-derived Leaders
SaGA-derived Followers
CD70
JAG1
ALCAM
IL13RA2
-Log10(p-value)
Log2(Fold change)
B
RNA-seq IL13RA2 expression
****
RPKM
P
F
L
C
Protein IL13RA2 expression
P
F
L
IL13Ra2
Actin
D
IL13RA2 Positive
IL13RA2 Negative
% Population
H1299
H1915
H2228
H460
H23
H1975
H549
H1755
SKMES1
CK5494
CK10151
CK9121

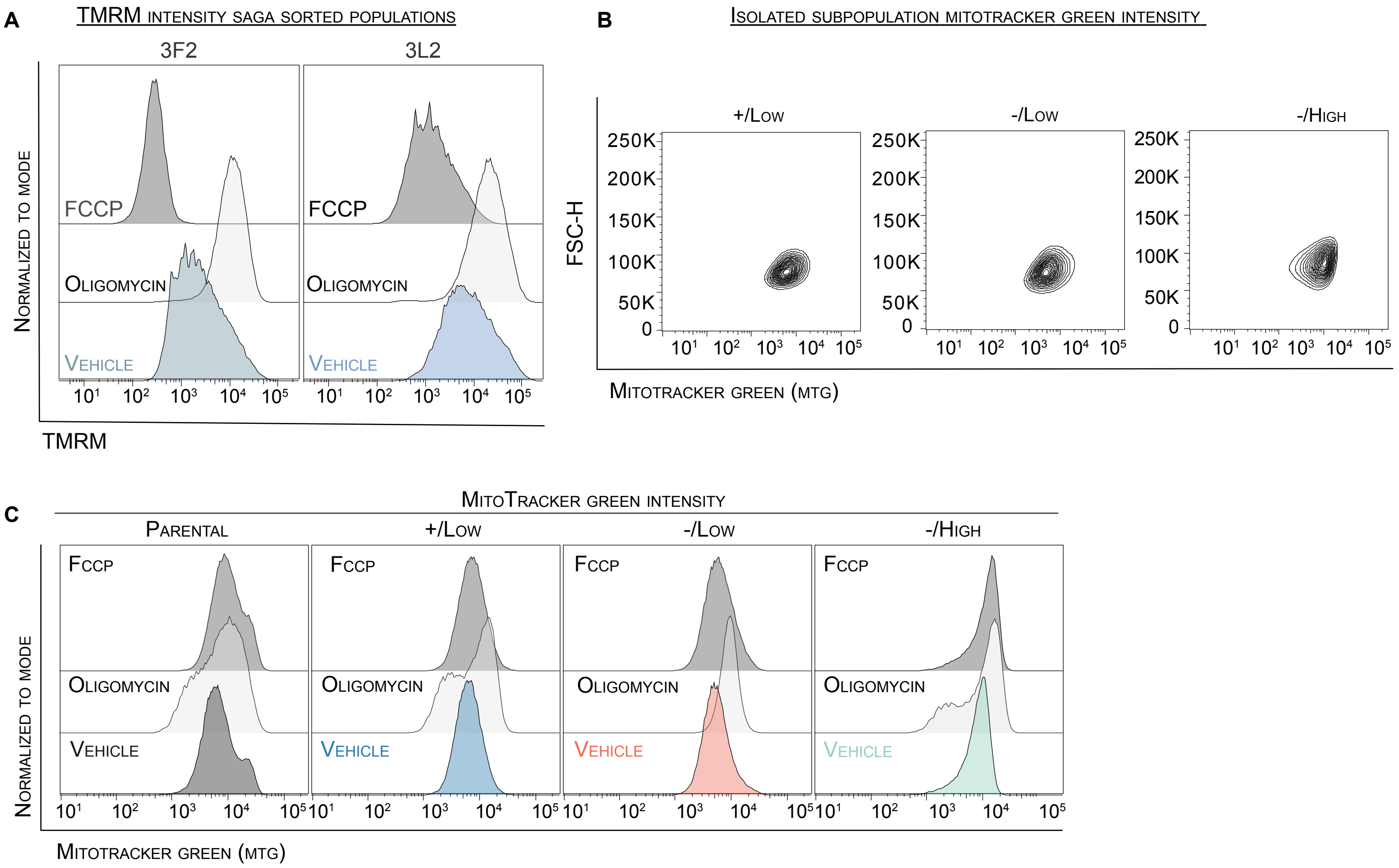
A
TMRM intensity SAGA sorted populations
3F2
3L2
FCCP
Oligomycin
Vehicle
Normalized to mode
TMRM
B
Isolated subpopulation MitoTracker green intensity
+/Low
-/Low
-/High
FSC-H
250K
200K
150K
100K
50K
0
MitoTracker green (MTG)
C
MitoTracker green intensity
Parental
+/Low
-/Low
-/High
Fccp
Oligomycin
Vehicle
Normalized to mode
MitoTracker green (MTG)

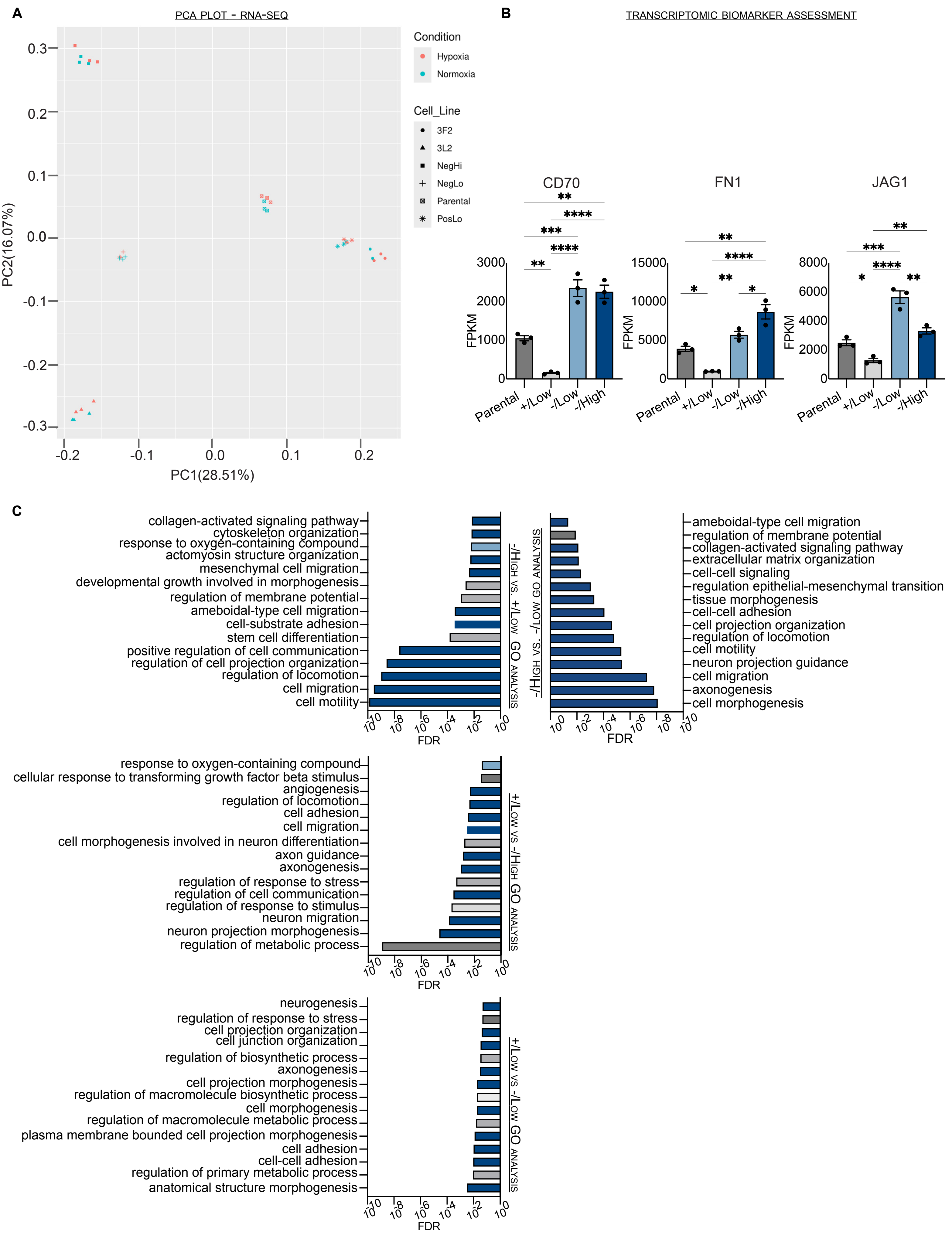
A
PCA PLOT - RNA-SEQ
Condition
Hypoxia
Normoxia
Cell_Line
3F2
3L2
NegHi
NegLo
Parental
PosLo
PC1(28.51%)
PC2(16.07%)
B
TRANSCRIPTOMIC BIOMARKER ASSESSMENT
CD70
FN1
JAG1
FPKM
Parental
+/Low
-/Low
-/High
C
collagen-activated signaling pathway
cytoskeleton organization
response to oxygen-containing compound
actomyosin structure organization
mesenchymal cell migration
developmental growth involved in morphogenesis
regulation of membrane potential
ameboidal-type cell migration
cell-substrate adhesion
stem cell differentiation
positive regulation of cell communication
regulation of cell projection organization
regulation of locomotion
cell migration
cell motility
-/HIGH VS. +/LOW GO ANALYSIS
FDR
ameboidal-type cell migration
regulation of membrane potential
collagen-activated signaling pathway
extracellular matrix organization
cell-cell signaling
regulation epithelial-mesenchymal transition
tissue morphogenesis
cell-cell adhesion
cell projection organization
regulation of locomotion
cell motility
neuron projection guidance
cell migration
axonogenesis
cell morphogenesis
-/HIGH VS. -/LOW GO ANALYSIS
response to oxygen-containing compound
cellular response to transforming growth factor beta stimulus
angiogenesis
regulation of locomotion
cell adhesion
cell migration
cell morphogenesis involved in neuron differentiation
axon guidance
axonogenesis
regulation of response to stress
regulation of cell communication
regulation of response to stimulus
neuron migration
neuron projection morphogenesis
regulation of metabolic process
+/LOW VS. -/HIGH GO ANALYSIS
neurogenesis
regulation of response to stress
cell projection organization
cell junction organization
regulation of biosynthetic process
axonogenesis
cell projection morphogenesis
regulation of macromolecule biosynthetic process
cell morphogenesis
regulation of macromolecule metabolic process
plasma membrane bounded cell projection morphogenesis
cell adhesion
cell-cell adhesion
regulation of primary metabolic process
anatomical structure morphogenesis
+/LOW VS. -/LOW GO ANALYSIS

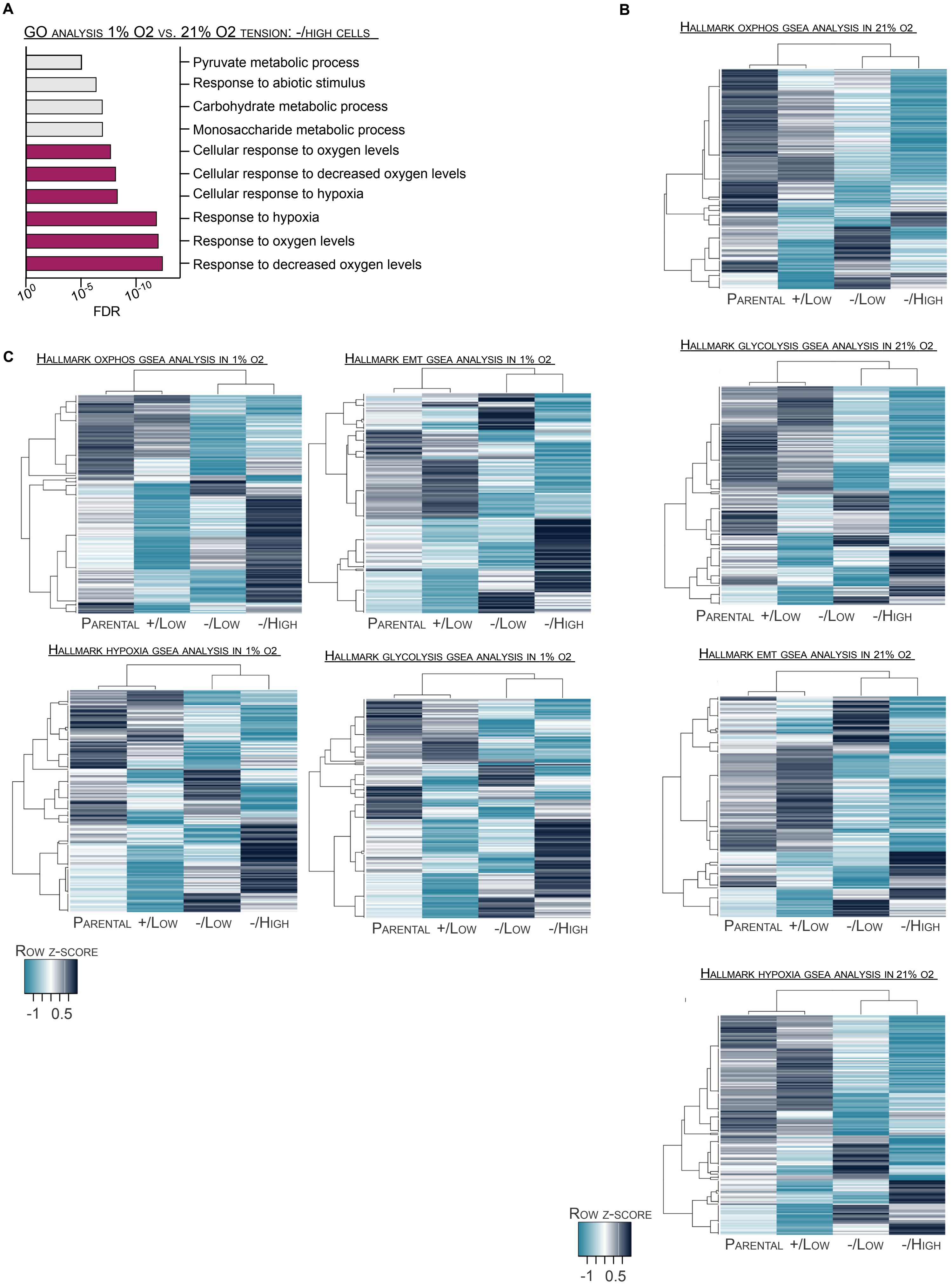
A
GO analysis 1% O2 vs. 21% O2 tension: -/high cells
Pyruvate metabolic process
Response to abiotic stimulus
Carbohydrate metabolic process
Monosaccharide metabolic process
Cellular response to oxygen levels
Cellular response to decreased oxygen levels
Cellular response to hypoxia
Response to hypoxia
Response to oxygen levels
Response to decreased oxygen levels
10^0
10^-5
10^-10
FDR
B
Hallmark oxphos GSEA analysis in 21% O2
Parental +/Low -/Low -/High
Hallmark glycolysis GSEA analysis in 21% O2
Parental +/Low -/Low -/High
Hallmark EMT GSEA analysis in 21% O2
Parental +/Low -/Low -/High
Hallmark hypoxia GSEA analysis in 21% O2
Parental +/Low -/Low -/High
Row z-score
-1 0.5
C
Hallmark oxphos GSEA analysis in 1% O2
Parental +/Low -/Low -/High
Hallmark EMT GSEA analysis in 1% O2
Parental +/Low -/Low -/High
Hallmark hypoxia GSEA analysis in 1% O2
Parental +/Low -/Low -/High
Hallmark glycolysis GSEA analysis in 1% O2
Parental +/Low -/Low -/High
Row z-score
-1 0.5

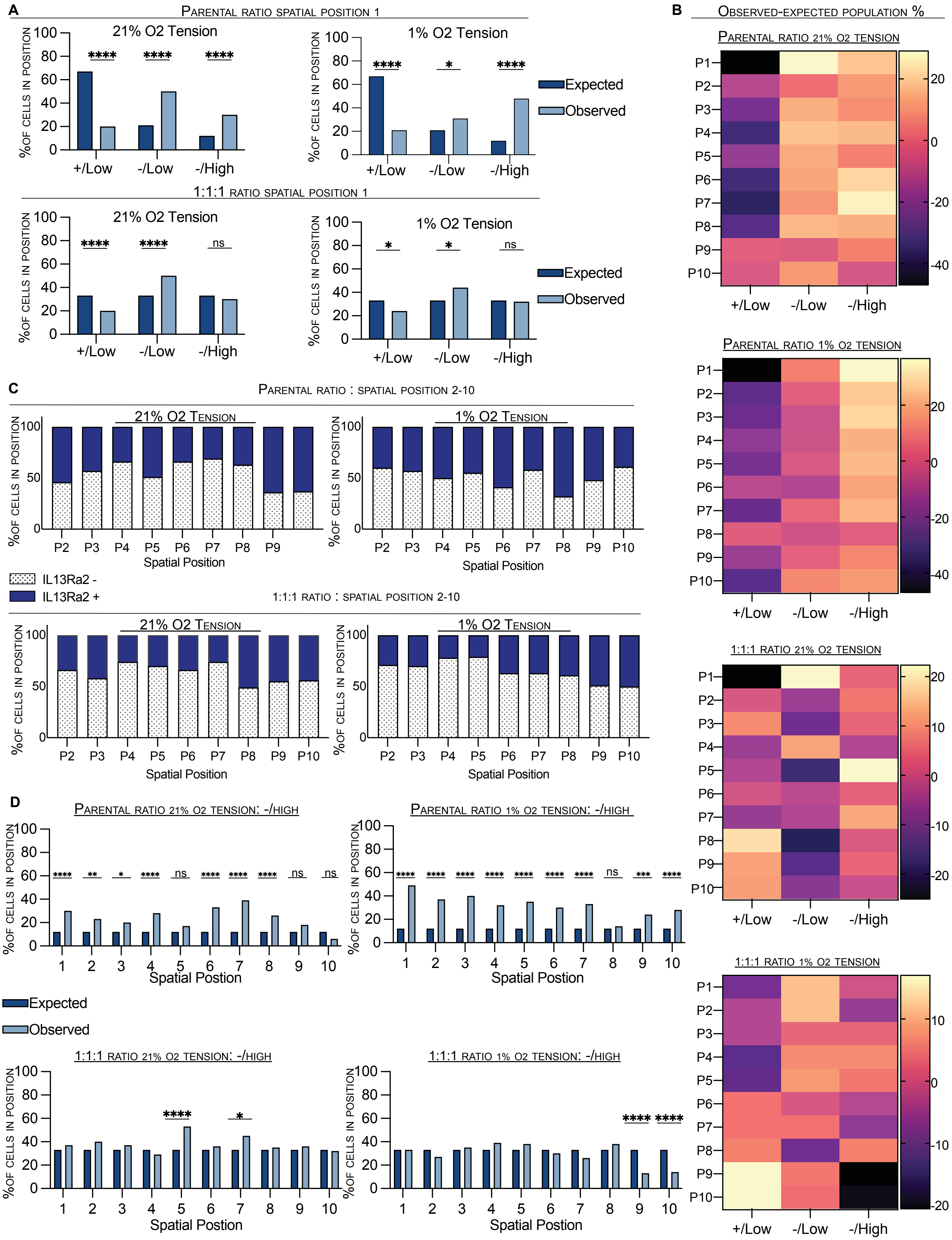

A
Parental ratio spatial position 1
21% O2 Tension
1% O2 Tension
%OF CELLS IN POSITION
+/Low
-/Low
-/High
Expected
Observed
1:1:1 ratio spatial position 1
B
Observed-expected population %
Parental ratio 21% O2 tension
Parental ratio 1% O2 tension
1:1:1 ratio 21% O2 tension
1:1:1 ratio 1% O2 tension
C
Parental ratio : spatial position 2-10
21% O2 Tension
1% O2 Tension
Spatial Position
IL13Ra2 -
IL13Ra2 +
1:1:1 ratio : spatial position 2-10
D
Parental ratio 21% O2 tension: -/high
Parental ratio 1% O2 tension: -/high
1:1:1 ratio 21% O2 tension: -/high
1:1:1 ratio 1% O2 tension: -/high
Spatial Postion

Figure S1. NSCLC cell lines and patient samples exhibit molecular and phenotypic heterogeneity, related to figure 1 (A) Volcano plot denoting differential gene expression for pair-wise comparison in cell surface markers in SaGA-derived leaders and followers analyzed based on RNA-sequencing data(n=3). (B) Bar chart quantifying IL13RA2 transcript levels in parental, SaGA-derived followers, and leaders. Data are represented as mean ±SEM. In order to assess significance, an ordinary one-way ANOVA with a Tukey's multiple comparisons test was used, $*p \le 0.05$, $**p \le 0.01$, $***p \le 0.001$, $****p \le 0.0001$ (n=3). (C) Immunoblots of whole cell lysate of parental, SaGA-derived followers, and leaders (n=3). (D) Bar chart quantifying the distribution of IL13RA2 cells in flow cytometry across NSCLC cell lines and patient-derived samples (n=2).

Figure S2. IL13RA2 and mitochondrial membrane potential characterize distinct subpopulations in NSCLC, related to figure 2 (A) Flow cytometry assessment of TMRM intensity in SaGA-derived leader and follower populations. At the time of TMRM staining, FCCP or Oligomycin were added to the samples and cells were analyzed by flow cytometry. Histograms normalized to the mode and representative of TMRM intensity are shown.(n=3). (B) Flow cytometry MitoTracker Green confirmation after subpopulation sorting(n=3). (C) Flow cytometry detection of mitochondria amount/load using MitoTracker Green. During MitoTracker Green staining, FCCP or Oligomycin were added to the samples and then the treated samples were analyzed by flow cytometry(n=2). Histograms normalized to the mode and representative of MitoTracker Green intensity are shown.

Figure S3. Stratification by IL13RA2 and mitochondrial membrane potential establishes phenotypically distinct subpopulations in NSCLC, related to figure 3 A) Principal component (PC) analysis plot of SaGA-derived leaders, SaGA-derived followers, +/Low,-/Low, and -/High, and H1299 parental cells cultured in normoxia (21% O_2 tension) and hypoxia (1% O_2 tension) based on RNA sequencing data (n=3).(B) Bar graphs quantifying CD70, FN1, and JAG1 transcript levels in parental, +/Low, -/Low, and -/High populations. Data represented as mean ±SEM. To assess significance, an ordinary one-way ANOVA with a Tukey's multiple comparisons test was used, $*p \le 0.05$, $**p \le 0.01$, $***p \le 0.001$, $****p \le 0.0001$. (n=3) (C) PANTHER GO pathway analysis for the most distinct proteins. p-value < 0.05, fold change >2, false discovery rate (FDR) <0.05. Dark blue indicates GO terms associated with migration/motility. Light blue indicates GO terms associated with oxygen/hypoxia.

Figure S4. Modulating oxygen tension results in distinct transcriptional and phenotypic profiles, related to figure 4 (A) PANTHER GO pathway analysis for the most distinct proteins after exposure to 1% O_2 tension for pair-wise comparison for -/High cells. p-value < 0.05, fold change >2, false discovery rate (FDR) <0.05. Pink indicates GO terms associated with oxygen/hypoxia. Three biological replicates were performed. (B) Heat map from RNA sequencing data using gene set enrichment analysis (GSEA). Scale denotes z scores from log_2- normalized expression counts of 'hypoxia,' 'oxidative phosphorylation,' 'epithelial-mesenchymal transition,' and 'glycolysis' hallmark gene sets from MSigDB in 21% O_2 samples.(n=3) (B) Gene set enrichment analysis as in (A) in 1% O_2 tension. (n=3)

Figure S6. 3D spatial positioning within composite populations is opportunistic and dependent on oxygen availability, related to figure 6 (A) Bar charts representing expected (dark blue) and observed (light blue) percentage of cells in spatial position 1. Significance was assessed using a chi-square test of independence. (B) Heat maps from spatial quantification (Figure 6C-H) for observed vs. expected population % differences. (C) Bar graphs quantifying the distribution of $IL13RA2^-$ (-/Low, -/High) and $IL13RA2^+$ cells in spatial position 2 through 10 in spheroids composed of a parental ratio in 21% and 1% O_2 tension. (D) Bar charts representing expected (dark blue) and observed (light blue) percentage of -/High cells in spatial position 1 through 10 in spheroids composed of a parental ratio and 1:1:1 ratio in 21% and 1% O_2 tension. Significance was assessed using a chi-square test of independence ≤ 0.05. Parental ratio in 21% O2 tension (n=3) and (N=39). Parental ratio in 1% O2 tension (n=3) and (N=43).1:1:1 ratio in 21% O2 tension(n=3) and (N=46). 1:1:1 ratio in 1% O2 tension(n=3) and (N=42).

Table S1. Statistical analysis of 1:1:1 ratio in 21% O_2 Tension, related to figure 6

		Spatial Position									
		P1	P2	P3	P4	P5	P6	P7	P8	P9	P10
+/Low	Exp	33	33	33	33	33	33	33	33	33	33
	Obs	7.9	34.2	42.1	26.3	28.9	33.3	25.8	51.7	44.4	44.0
	P-value	5.8E-08	8.3E-01	6.1E-02	1.3E-01	3.4E-01	9.2E-01	1.1E-01	9.0E-05	1.8E-02	2.3E-02
-/Low	Exp	33	33	33	33	33	33	33	33	33	33
	Obs	55.3	26.3	21.1	44.7	15.8	30.3	29.0	13.8	18.5	24.0
	P-value	2.8E-06	1.3E-01	9.1E-03	1.5E-02	1.8E-04	5.1E-01	3.5E-01	3.1E-05	1.6E-03	4.6E-02
-/High	Exp	33	33	33	33	33	33	33	33	33	33
	Obs	36.8	39.5	36.8	28.9	55.3	36.4	45.2	34.5	37.0	32.0
	P-value	4.6E-01	1.9E-01	4.6E-01	3.5E-01	2.8E-06	5.1E-01	1.1E-02	7.9E-01	4.3E-01	7.6E-01

Legend table 1: Expected (Exp) percentages, observed(Obs) percentages, and p-values from spatial position quantification for the 1:1:1 ratio in 21% O_2 tension for +/Low,-/Low, and -/High cells, significance was assessed using chi-squared test of independence. Conditions in which $p<0.05$ are highlighted in red. 1:1:1 ratio in 21% O2 tension(n=3) and (N=46).

Table S2. Statistical analysis of 1:1:1 ratio in 1% O_2 Tension, related to figure 6

		Spatial Position									
		P1	P2	P3	P4	P5	P6	P7	P8	P9	P10
+/Low	Exp	33	33	33	33	33	33	33	33	33	33
	Obs	23.5	29.4	29.4	21.2	21.9	37	37	38.5	50	50
	P-value	3.6E-02	4.0E-01	4.0E-01	9.6E-03	1.5E-02	4.3E-01	4.3E-01	2.7E-01	3.8E-04	3.8E-04
-/Low	Exp	33	33	33	33	33	33	33	33	33	33
	Obs	44.1	44.1	35.3	39.4	40.6	33.3	37	23.1	37.5	36.4
	P-value	2.2E-02	2.2E-02	6.7E-01	1.9E-01	1.2E-01	9.3E-01	4.3E-01	2.8E-02	3.7E-01	5.1E-01
-/High	Exp	33	33	33	33	33	33	33	33	33	33
	Obs	32.4	26.5	35.3	39.4	37.5	29.6	25.9	38.5	12.5	13.6
	P-value	8.2E-01	1.4E-01	6.7E-01	1.9E-01	3.7E-01	4.3E-01	1.1E-01	2.7E-01	8.9E-06	2.6E-05

Legend table 2: Expected (Exp) percentages, observed(Obs) percentages, and p-values from spatial position quantification for the 1:1:1 ratio in 1% O_2 tension for +/Low,-/Low, and -/High cells, significance was assessed using chi-squared test of independence. Conditions in which $p<0.05$ are highlighted in red. 1:1:1 ratio in 1% O2 tension(n=3) and (N=42).

Table S3. Statistical analysis of parental ratio in 21% O_2 Tension, related to figure 6

		Spatial Position									
		P1	P2	P3	P4	P5	P6	P7	P8	P9	P10
+/Low	Exp	67	67	67	67	67	67	67	67	67	67
	Obs	20.0	55.0	42.5	33.3	48.6	33.3	30.4	36.8	64.7	62.5
	P-value	1.6E-23	1.1E-02	1.2E-06	7.7E-13	9.1E-05	8.5E-13	7.9E-15	1.5E-10	6.3E-01	3.3E-01
-/Low	Exp	22	22	22	22	22	22	22	22	22	22
	Obs	50.0	22.5	35.0	38.5	34.3	33.3	30.4	36.8	17.6	31.3

	P-value	1.1E-12	7.1E-01	3.5E-04	1.7E-05	1.1E-03	2.5E-03	2.1E-02	1.0E-04	4.1E-01	1.2E-02
-/High	Exp	12	12	12	12	12	12	12	12	12	12
	Obs	30.0	22.5	20.0	28.2	17.1	33.3	39.1	26.3	17.6	6.3
	P-value	3.0E-08	1.2E-03	1.0E-02	6.2E-07	1.2E-01	5.4E-11	7.2E-17	1.1E-05	8.4E-02	7.9E-02

Legend table 3: Expected (Exp) percentages, observed(Obs) percentages, and p-values from spatial position quantification for the parental ratio in 21% O_2 tension for +/Low,-/Low, and -/High cells, significance was assessed using chi-squared test of independence. Conditions in which $p<0.05$ are highlighted in red. Parental ratio in 21% O2 tension (n=3) and (N=39).

Table S4. Statistical analysis of parental ratio in 1% O_2 Tension, related to figure 6

		Spatial Position									
		P1	P2	P3	P4	P5	P6	P7	P8	P9	P10
+/Low	Exp	67	67	67	67	67	67	67	67	67	67
	Obs	20	40	42.9	50	44.8	59.3	41.7	68.2	52.4	38.9
	P-value	1.6E-23	9.4E-09	3.0E-07	3.0E-04	2.3E-06	1.0E-01	7.4E-08	8.0E-01	1.9E-03	2.3E-09
-/Low	Exp	22	22	22	22	22	22	22	22	22	22
	Obs	31.4	22.9	17.1	17.6	20.7	11.1	25	18.2	23.8	33.3
	P-value	1.1E-02	6.4E-01	3.4E-01	4.0E-01	9.4E-01	1.5E-02	3.3E-01	4.9E-01	4.9E-01	2.5E-03
-/High	Exp	12	12	12	12	12	12	12	12	12	12
	Obs	48.6	37.1	40	32.4	34.5	29.6	33.3	13.6	23.8	27.8
	P-value	2.0E-29	1.1E-14	6.9E-18	3.4E-10	4.4E-12	6.1E-08	5.6E-11	6.2E-01	2.8E-04	1.2E-06

Legend table 4: Expected (Exp) percentages, observed(Obs) percentages, and p-values from spatial position quantification for the parental ratio in 1% O_2 tension for +/Low,-/Low, and -/High cells, significance was assessed using chi-squared test of independence. Conditions in which $p<0.05$ are highlighted in red. Parental ratio in 1% O2 tension (n=3) and (N=43).
